# Mental health training programmes for non-mental health trained professionals coming into contact with people with mental ill health: a systematic review of effectiveness

**DOI:** 10.1186/s12888-017-1356-5

**Published:** 2017-05-25

**Authors:** Alison Booth, Arabella Scantlebury, Adwoa Hughes-Morley, Natasha Mitchell, Kath Wright, William Scott, Catriona McDaid

**Affiliations:** 1 York Trials Unit, Department of Health Sciences, Faculty of Science, University of York, Helslington, York YO10 5DD UK; 2 Centre for Reviews and Dissemination, University of York, Helslington, York UK; 3 North Yorkshire Police, Newby Wiske, UK

**Keywords:** Training, Mental health, Police, Systematic review

## Abstract

**Background:**

The police and others in occupations where they come into close contact with people experiencing/with mental ill health, often have to manage difficult and complex situations. Training is needed to equip them to recognise and assist when someone has a mental health issue or learning/intellectual disability. We undertook a systematic review of the effectiveness of training programmes aimed at increasing knowledge, changing behaviour and/or attitudes of the trainees with regard to mental ill health, mental vulnerability, and learning disabilities.

**Methods:**

Databases searched from 1995 onwards included: ASSIA, Cochrane Central Register of Controlled Clinical Trials (CENTRAL), Criminal Justice Abstracts, Embase, ERIC, MEDLINE, PsycINFO, Social Science Citation Index. Courses, training, or learning packages aimed at helping police officers and others who interact with the public in a similar way to deal with people with mental health problems were included. Primary outcomes were change in practice and change in outcomes for the groups of people the trainees come into contact with. Systematic reviews, randomised controlled trials (RCTs) and non- randomised controlled trials (non-RCTs) were included and quality assessed. In addition non-comparative evaluations of training for police in England were included.

**Results:**

From 8578 search results, 19 studies met the inclusion criteria: one systematic review, 12 RCTs, three prospective non-RCTs, and three non-comparative studies.

The training interventions identified included broad mental health awareness training and packages addressing a variety of specific mental health issues or conditions. Trainees included police officers, teachers and other public sector workers.

Some short term positive changes in behaviour were identified for trainees, but for the people the trainees came into contact with there was little or no evidence of benefit.

**Conclusions:**

A variety of training programmes exist for non-mental health professionals who come into contact with people who have mental health issues. There may be some short term change in behaviour for the trainees, but longer term follow up is needed. Research evaluating training for UK police officers is needed in which a number of methodological issues need to be addressed.

**Trial registration:**

**Protocol registration number:** PROSPERO: CRD42015015981.

**Electronic supplementary material:**

The online version of this article (doi:10.1186/s12888-017-1356-5) contains supplementary material, which is available to authorized users.

## Background

Police officers fulfil numerous roles bringing them into close contact with the general public including people with mental ill health [1]. In the UK the police are signatories of the Mental Health Crisis Care Concordat, a multi-agency initiative aimed at improving outcomes for people experiencing mental health crisis [2]. The Concordat recognises the pivotal role played by the police in identifying and deciding on the most appropriate course of action in situations involving individuals with mental ill health. However, police officers are not experts in mental ill health, and often have to manage complex situations with insufficient training. In the UK, gaps in knowledge have been identified, particularly around police officers’ understanding of Section 136 of the Mental Health Act 1983, which gives them the power to remove from public places anyone who appears to be suffering from mental disorder and take them to a place of safety [1, 3]. Additionally, police officers were not always aware of their responsibilities, even when guidance was provided. The review identified online learning as the main form of training tool for police officers; however there were variations in who had completed the training [1]. Training is a priority for the police, however, the best approach to train officers to respond to people with mental health problems remains unclear. We undertook this systematic review to identify evidence of effectiveness of training approaches to inform the development of a mental health training package for police officers which is currently being assessed in a cluster RCT comparing the new training package with routine training (ISRCTN registry trial ID ISRCTN11685602).

While our main interest was training for police officers, preliminary searches suggested there may be limited studies available specifically in the police. We therefore widened the scope to include non-mental health professionals who may interact with the public in a similar way to the police. Being able to identify problems, deal with situations or refer for professional assistance is expected of people such as teachers and case workers, despite their non-mental health training. It was anticipated that widening the scope would capture a broader range of training approaches that would be relevant in the police setting. We looked at the international literature but as our aim was to inform development of an intervention in the UK, we included a wider range of study designs from the UK to provide context.

We aimed to:

1. Evaluate the evidence on the effectiveness: of training programmes and/or training resources aimed at increasing knowledge and/or changing behaviour or attitudes of the trainees with regard to mental ill health, mental vulnerability, and learning disabilities; and of satisfaction with training and barriers and facilitators to effective training.

2. Identify methods used for evaluating the impact of training interventions.

## Methods

The review followed an a priori protocol: details including minor amendments are available in PROSPERO record CRD42015015981.

### Selection criteria

*Participants/population:* specific mental health training programmes targeted at police officers; other police staff who come into contact with the public; members of the criminal justice system; non-mental health trained health professionals working in acute health care, including paramedics; people working in education; any other professions, responsible organisations or mental health charities who interact with the public. We excluded basic training delivered to trainees or newly appointed staff in the police force, but included evaluations of additional training. Studies from Organisation for Economic Co-operation and Development (OECD) countries only were included.

*Intervention:* Training aimed at increasing knowledge of mental health, mental vulnerability, or learning disabilities of members of the public and/or changing attitudes and/or improving their skills in dealing with people with mental health problems in their role, was included. Any courses, training, learning packages or other training resources delivered by any method, for example face-to-face; self-directed; team based or web-based programmes were included. This includes role modelling, reflection, online or mobile phone apps or written materials. Training could focus on the mental health of children, young people and adults.

We used the UK Mental Health Act 2007 definition of mental ill health to encompass, “any disorder or disability of the mind”, [3] whether or not a formal diagnosis had been made. Learning disabilities (also called intellectual disabilities) were included in line with the National Policing Improvement Agency guidance on responding to people with mental ill health or learning disabilities [4].

*Comparator(s):* no-training, usual practice or comparison between different training approaches, for example class room based training vs on-line training.

*Outcomes:* we were interested in seven possible outcomes of training interventions [5–8]. These were classified into two primary outcomes: change in practice (evaluation of behaviour); and change in outcomes for the groups of people the trainees come into contact with (evaluation of results). The five secondary outcomes were: satisfaction with training (evaluation of reaction), change in attitude towards the importance of mental health, change in confidence, change in knowledge and change in skills (evaluation of learning).

*Types of study included:* systematic reviews (that reported their inclusion/exclusion criteria, searched at least one database, provided study details and/or a quality assessment, and synthesised included studies); and RCTs. We included non-RCTs and observational studies without a control group for the police group only, to identify relevant interventions to inform development of the training package. Published and unpublished audits and evaluations of police training in mental health in England and Wales were included.

Qualitative studies of views and experiences of training, and barriers and facilitators to implementation were included to capture satisfaction with training delivery and implementation.

### Searches

An information specialist searched the following bibliographic databases for English language studies from 1995 onwards: ASSIA, Cochrane Central Register of Controlled Clinical Trials (CENTRAL), Criminal Justice Abstracts, Embase, ERIC, MEDLINE, PsycINFO, Social Science Citation Index. The search strategies are available in Additional file 1. We also scanned the complete list of Campbell Reviews and the Register of Studies produced by the Cochrane Effective Practice and Organisation of Care Group. Restricting the searches to the last 20 years took into account the changing legislation attitudes and awareness of mental health.

We checked the reference lists of included studies and of papers that had cited the included papers. The websites of major UK mental health charities were searched (details in Additional file 1). We also searched PROSPERO and ISRCTN for relevant ongoing or completed but not yet published reviews and trials.

Police training officers in England and Wales were asked for their help in identifying published or unpublished audits or evaluations on the impact of mental health training delivered to police officers and/or staff.

### Study selection and data collection

Titles and abstracts of all studies were screened independently by two researchers. Full papers were assessed for inclusion independently by two researchers and at both stages discrepancies were resolved through discussion.

Data extraction forms were developed in Microsoft Excel 2010 and piloted. Data extracted included details of study design (setting, aims, unit of allocation, inclusion criteria, recruitment method etc.), intervention and comparator (type of training, method of delivery, presentation elements, length of training, where delivered, aims of training), and evaluation methods (timing, outcomes measured). Data extraction was undertaken by one researcher and checked by a second; discrepancies were resolved by discussion with a third researcher.

Interventions were classified as didactic if they were exclusively made up of lectures (e.g. presenter determines content, organisation and pace), interactive if they included active participation by the trainees (e.g. case studies, role-play, group work), and mixed if both were used [5–8].

### Risk of bias (quality) assessment

The ROBIS tool [9] was used to assess the risk of bias of the systematic review; the Cochrane Risk of Bias tool for RCTs [10]; the Newcastle-Ottawa Quality Assessment Scale for Cohort Studies [11]; and the National Institutes for Health tool for studies without a control group [12]. Assessment was undertaken by one researcher and independently checked by a second, with discrepancies resolved by discussion.

### Strategy for data synthesis

We performed a narrative synthesis as a meta-analysis was not feasible due to the substantial differences in interventions and methodological approaches in the included studies. The small number of studies precluded subgroup analysis of short courses as planned in our protocol. Identified qualitative studies provided insight into the barriers, facilitators and perceived impact of training and are presented in a separate paper.

## Results

**Fig. 1:**
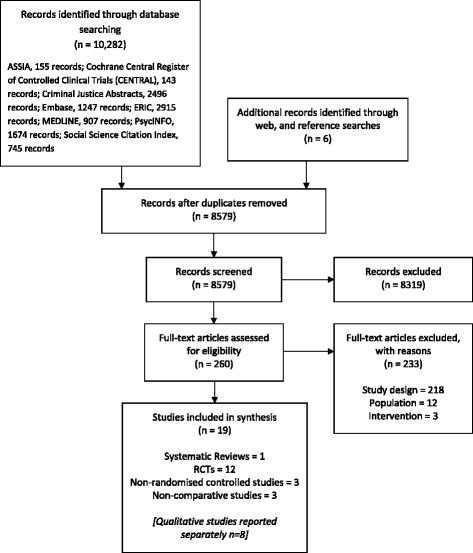
Flow of studies through review process

The search strategies and allied searching identified 8579 references (after deduplication): these were loaded into EndNote ×7 (Thomson Reuters, CA, USA) bibliographic software. Figure 1 shows the flow of studies through the review process. We identified 19 studies for inclusion, and a further eight qualitative studies which are reported separately. Additional file 2 lists the excluded studies.

One systematic review [13], 12 RCTs [14–25] and three prospective non-RCTs of police training [26–28], were included. In addition we included three non-comparative evaluations of mental health related training interventions specifically for the police in England [29–31]. A summary of the characteristics of the included studies is provided in Table 1.

**Table 1:** Characteristics of included studies

Study	Study design	Training evaluated	Participants	Comparator	Number of participants in study	Number of participants in analysis
Country	Type of evaluation				Number of clusters	
Police officers: Interventions with a broad mental health focus						
Compton (2008) [13]	Systematic review	*Crisis Intervention Team programs (CIT)*	Police officers	No comparators specified however the broad scope was “research on CIT”	12 studies were included in the review	Unclear. No summary table of studies provided. The studies are presented in a narrative synthesis. Some studies reported number of participating police officers and others number of incidents (*n* = 929 police officers for the 6 studies of officer level outcomes)
USA	Provide a systematic summary and critical analysis of research on crisis intervention teams	Evaluations, surveys, or outcome studies conducted to examine specific questions or test hypotheses were included				
		Reports of descriptive statistics were excluded				
		Searches included: databases (MEDLINE, PsycINFO, and databases of criminal justice, criminology and sociology abstracts), grey literature searches, and reference checking				
Forni et al. (2009) [29]	Non-comparative study	*Mental health awareness training* to improve communication and understanding; explore common problems and solutions; understand how the Mental Health Unit functions; how MH staff handle violence and aggression; understand common MH problems	Police officers	Not applicable	364 received training	280 returned evaluation forms
England	Post training evaluation form					
Hansson & Markstrom (2014) [27]	Prospective non-RCT	An anti-stigma intervention aimed at improving knowledge behaviour and attitudes towards people with mental illness	Police officers	No training	120	105 (46 intervention 59 control)
	Pre and post intervention questionnaire. 6 month follow up of intervention group					
Sweden						
Herrington & Pope (2013) [28]	Prospective non-RCT	*Mental Health Intervention Team (MHIT):* training uniformed officers as specialists to respond to individuals with an apparent MH concern	Front line police officers (constables, senior constables, sergeants)	No training	185	Unclear
	Training group: pre, 2 months and 18 months post intervention questionnaires					
Australia						
	Control group: questionnaire at 18 months only. Routinely collected data, semi-structured interviews, observational data, focus groups					
Norris & Cooke (2000) [30]	Non-comparative study	Training package developed by MH professionals. Aims to provide police officers with understanding, skills and awareness, and aid the management of mental disorder or mental illness	Police officers	Not applicable	132 officers trained	57 responses (43%)
England	Immediate post training evaluation and retrospective survey					
Pinfold et al. (2003) [31]	Non-comparative study	*Mental health awareness training* developed in house. Aim to reduce negative stereo-typing and discriminatory actions	Police officers	Not applicable	232 were eligible to take part; 163 were trained	119 provided follow-up data
England	Pre and 4 weeks post training surveys					
Rafacz (2012) [17]	RCT	Online anti-stigma video aimed at changing attitudes, reduce stigma and increase empathy	Campus police officers	Alternative training	91	91^d^
USA	Multiple pre and post intervention surveys.					
Police officers: Interventions with a specific mental health focus						
Bailey et al. (2001) [26]	Prospective non-RCT	Awareness raising training on intellectual disabilities in general	Police officer trainees	No training	62	57
						(27 intervention 30 control)
Northern Ireland	Pre and post intervention questionnaire					
Teagardin et al. (2012) [15]	Cluster RCT	*Law Enforcement: Your Piece to the Autism Puzzle.* Aims to help the recognition and identification of and attitudes towards people with Autism Spectrum Disorders (ASD)	“in the field” law enforcement officers (e.g. a patrol officer or detective)	Waiting list	82	81^e^
					Unclear	
USA	Pre and post intervention survey					
Other non-mental health trained professionals: Interventions with a broad mental health focus						
Dorsey et al. (2012) [25]	Cluster RCT	*Project Focus*: training and consultation model to improve knowledge of evidence based practices and ability to classify MH problems and referral options	Child welfare caseworkers	Waiting list	67	44
USA	Questionnaire and a vignette based knowledge test pre and post intervention				4 offices	
Jorm et al. (2010) [23]	Cluster RCT	A modified version of the *Youth Mental Health First Aid* course. Aims to improve knowledge, attitudes, confidence and behaviour for dealing with students MH problems	Teachers	Waiting list	327	327
Australia	Pre and post intervention and 6-month follow up questionnaire					
					14 schools	
Lipson et al. (2014) [21]	Cluster RCT	*Mental Health First Aid.* Aims to improve attitudes and increase knowledge and self-efficacy to manage MH issues	Resident advisors	Usual practice	254	2543
					32 colleges and universities	
USA	Surveys 2–3 months pre and post training					
Svensson & Hansson (2014) [16]	RCT	*Mental Health First Aid* (translated and modified) To improve MH literacy and give skills to provide help to people with MH problems	Public sector staff	Waiting list	406	406
	Questionnaires and vignette 1 month pre intervention; 6 months post intervention and 2 year follow-up					
Sweden						
Thombs et al. (2015) [14]	Cluster RCT	*Peer Hero Training Programme.* Aims to improve skills, knowledge & confidence in managing responses to MH and other situations	Resident assistants	Usual practice	652	566^c^
USA	On-line survey at baseline, follow-ups at 3, 7 and 9 months				8 campuses	
Other non-mental health trained professionals: Interventions with a specific mental health focus						
Hart & More (2013) [24]	RCT	Audio-visual synchronous podcast of information related to Autism Spectrum Disorder (ASD) with the aim of improving knowledge	Trainee teachers	Alternative training	79	79
USA	Pre and post intervention survey					
Kolko et al. (2012) [22]	RCT	*Alternatives for Families: A Cognitive–Behavioral Therapy (AF-CBT).* Aims to provide skills to use AF-CBT when working with families with physical forces, aggression or abuse of children	Community practitioners	Usual practice	182	128
USA	Questionnaires completed at baseline, 6, 12 and 18 months					
McVey et al. (2008) [20]	Cluster RCT	*The student body*: *promoting health at any size an online programme.”* Aims to help prevent the onset of disordered eating through the promotion of positive body image	Teachers and public health professionals	Waiting list	167	85^b^
Canada	Pre and 60 days post intervention questionnaire				37 schools and their public health agencies (number unclear)	
Moor et al. (2007) [19]	RCT	Educational package on recognition, identification and attitudes towards adolescent depression	Teachers	Waiting list	151	151
Scotland	Pre and post training questionnaire					
Ostberg & Rydell (2012) [18]	RCT	A modified version of *Barkley’s parent training programme.* Aims to provide tools and strategies to help children with Attention Deficit Hyperactivity Disorder (ADHD)	Parents and teachers	Waiting list	147	129^a^
Sweden	Pre and post intervention and 3-month follow up questionnaire					

^a^For most but not all analyses. ^b^Calculated from % of non-completers. ^c^The authors state results for 566 are reported, but Fig. 1 shows total of 565 at baseline measures. ^d^91 completed initial part; 52 completed follow up part; data were imputed for missing responses. ^e^ One control participant withdrew following second pre-test

The studies included in the systematic review were all conducted in the USA. Eight of the 19 primary studies included took place in the USA, three in Sweden, three in England, two in Australia, and one each in Canada, Scotland and Northern Ireland. Participants included teachers, public health professionals, university resident advisors, community practitioners, public sector staff, and case workers. Law enforcement participants were trainee, probationary, university campus, and front line police officers.

Where details of the training venue were reported, this was generally in a training facility environment at participants’ place of work: two of the studies held the training in specialist mental health units. Where reported, the number receiving training in a session ranged from 15 to 42 per group. Intervention delivery classed as short (over 1 day or less) ranged from 15 min to 4 h; and longer delivery times ranged from 12 h over 2 days to 47 h with a 1.5 to 2 h annual booster.

### Evaluation of outcomes

A summary of the measures used for each of the seven outcome categories for this review is provided in Table 2. The main method of evaluation in the included studies was participant completed questionnaire. Reporting of details of the instrument(s) used was generally incomplete. Most studies used a combination of questionnaires to evaluate different aspects of interest. Some validated instruments were used, but most were then modified by the exclusion of questions and/or addition of new questions [20–22, 31]. Many questionnaires were developed by the researchers specifically for their study [14–16, 19–21, 23–25, 29, 30]; most undertook some form of check for internal consistency [14, 19, 20, 22, 24–26].

**Table 2:** Outcome measures used

	Primary outcomes		Secondary outcomes				
Study	Change in practice	Change in outcomes for the groups of people the trainees come into contact with	Satisfaction with training:	Change in attitude towards the importance of mental health	Change in confidence	Change in knowledge:	Change in skills - change in behaviour/ application of skills:
*Police officers: Interventions with a broad mental health focus*							
Forni et al. (2009) [29]	Not measured	Not measured	Feedback form evaluated quality of presentation and content	Form evaluated quality of presentation and content	Not measured	Form evaluated quality of presentation and content	Form evaluated quality of presentation and content
Hansson & Markstrom (2014) [27]	Not measured	Not measured	Not measured	Attitudes: Community Attitudes towards Mental Illness. Validated. 20 items. Score: 6-point Likert scale.	Not measured	Mental Health Knowledge Scale. Validated 12 items, scored on 1–5 scale	Not measured
				Behaviour: Reported and Intentional Behaviour Scale: Validated. 8 items: 4 scored Yes/No and 4 on 1–5 scale			
Herrington & Pope (2013) [28]	Surveys. Number of items and validation not reported	Surveys. Number of items and validation not reported	Surveys. Number of items and validation not reported	Not measured	No comparative data reported	Not measured	Surveys. Number of items and validation not reported
Norris & Cooke (2000) [30]	Questionnaire developed by study team	Not measured	Not measured	Not measured	Not measured	Not measured	Not measured
Pinfold et al. (2003) [31]	Modified Community Attitudes towards Mental Illness (CAMI) and World Psychiatric Association (WPA 2000) questionnaires. Scored on 5 point likert scale	Not measured	One page feedback form	Modified CAMI and WPA 2000 questionnaires. Scored on 5 point likert scale	Modified CAMI and WPA 2000 questionnaires. Scored on 5 point likert scale	Modified CAMI and WPA 2000 questionnaires. Scored on 5 point likert scale	Modified CAMI and WPA 2000 questionnaires. Scored on 5 point likert scale
Rafacz (2012) [17]	Not measured	Not measured	Not measured	Modified Attribution Questionnaire. 31 questions, response on a 5-point Likert scale	Not measured	Not measured	Not measured
Police officers: Interventions with a specific mental health focus							
Bailey et al. (2001) [26]	Not measured	Not measured	Not measured	Attitudes towards Mental Retardation and Eugenics questionnaire. Validated. 32 items measured on a Likert scale. Score range 32–192	Not measured	Not measured	Not measured
Teagardin et al. (2012) [15]	Not measured	Not measured	Not measured	Not measured	2 of a 12 item questionnaire developed by study team: scored on Likert scale	10 of a 12 item questionnaire developed by study team: scored on Likert scale	Not measured
Other non-mental health trained professionals: Interventions with a broad mental health focus							
Dorsey et al. (2012) [25]	54 item questionnaire developed by study team	Not measured	Not measured	Not measured	Not measured	Vignette-based knowledge test developed by study team	Vignette-based knowledge test developed by study team
Jorm et al. (2010) [23]	Not measured	Strengths & Difficulties Questionnaire: validated measure of 25 items: score range 0–3	Not measured	Vignette-based knowledge test developed by study team. Non-validated	Two item questionnaire developed by the study team. Non-validated. Score range 0–5	21 item questionnaire developed by the study team. Non-validated. Score range 0–3	4 item questionnaire developed by the study team. Non-validated. Score range 0–4
Lipson et al. (2014) [21]	Study team produced categorical measure of the number of residents with whom Resident Advisors discussed mental health issues (0, 1, 2–3, or 4+);	The K6 scale for psychological distress. Validated scale. Score range: 0 to 24	Not measured	3 item measure of personal stigma adapted from the existing Discrimination-Devaluation Scale. Score range: 0–5	Measure used was not clearly reported	Non-validated question developed by the researchers. 5 response categories	Non-validated 6 items related to self-perceived gatekeeper skills.
Svensson & Hansson (2014) [16]	Pre-existing Questionnaires with vignettes. Vignettes scored max. 5 or 6	Not measured	Not measured	Pre-existing Social distance scale. 6 items, scored on a 1–3 scale.	Pre-existing Questionnaires with vignettes. Vignettes scored max. 5 or 6	Non-validated Questionnaire developed by study team. 16 items, response scale 0–2. Score range 0–16	Pre-existing Questionnaires with vignettes. Vignettes scored max. 5 or 6
Thombs et al. (2015) [14]	12 item survey developed by study team measured the number of first aid efforts made over the previous 30 days	Not measured	Not measured	Not measured	Referral efficacy, scored on a 5-point Likert scale	Not measured	Not measured
Other non-mental health trained professionals: Interventions with a specific mental health focus							
Hart & More (2013) [24]	Not measured	Not measured	Not measured	Not measured	Not measured	Non-validated questionnaire, with ten open-ended questions: score range 0–3	Not measured
Kolko et al. (2012) [22]	35 item AF-CBT implementation measure (AIM). Scores rated on a 5-point Likert-type scale	Not measured	13-item training evaluation developed by The National Child Trauma Stress Network. 11 Items scored on a 1–5 Likert-type scale; 2 items open ended	Not measured	Not measured	25-item CBT Knowledge Questionnaire. Multiple-choice: 4 or 5 response choices.	35 item AF-CBT implementation measure (AIM). Scores rated on a 5-point Likert-type scale
McVey et al. (2008) [20]	Not measured	Not measured	A 24 item non-validated questionnaire developed by study team. a 4-point, forced-choice scale	6-item questionnaire developed by study team, rated on a 4-point Likert scale	6-item subscale from existing validated “Free to Be Me” survey, adapted for teachers. Score: 4-point Likert scale	Validated survey “Free to Be Me” (modified); in-house survey; items, from Perceived Media Influences Sub-Scale. Scored on a 4/5-point Likert scale	6-item subscale from existing validated “Free to Be Me” survey, adapted for teachers. Score: 4-point Likert scale
Moor et al. (2007) [19]	Percentage of pupils reported as depressed by teachers, using lists of class cohort	Not measured	Not measured	Not measured	Attitudes Questionnaire designed by the research team. This was piloted however unclear to what extent it was validated. 30mins to complete	Not measured	Lists of class cohort
Ostberg & Rydell (2012) [18]	Not measured	ADHD Rating Scale 0–4; DSM-IV scale 0–4; Strengths and Difficulties Questionnaire validated, 20-items, scale 1–5	Described by authors as measured, but how was not reported	Not measured	Not measured	Not measured	Not measured

N.B. Number of items and scoring are included in the table where they were available. None of the studies included an economic evaluation or reported resource use or costs

Three of the RCTs used vignettes combined with a questionnaire to evaluate changes in practice [16], attitude [23], confidence [16], knowledge [25], and behaviour [16, 25]. Other measures used were basic rating scales, for example a categorical measure of the number of residents with whom university resident advisors interacted [21]. None of the included studies undertook an economic evaluation or reported resource use or costs.

### Risk of bias within studies

A summary risk of bias table is provided in Additional file 3. Overall the included studies were not well reported, in particular, reporting omissions made it difficult to extract or calculate an intervention effect and 95% confidence interval which would have allowed us to report results across studies.

The systematic review was at high risk of bias [13]. It was unclear whether all the appropriate studies were included in the review, particularly as the authors reported on several studies not identified by their searches. The included studies were not quality assessed and little detail was provided to allow interpretation of the results.

Overall the risk of bias in the RCTs was judged to be unclear because details were not reported, or high risk due to issues with randomisation, blinding or incomplete outcome data [14–25]. The risk of bias in the non-RCTs was generally unclear, mainly because of inadequate reporting of the methods [26–28]. The quality rating for one of the non-comparative studies was good [31] and the other two had reporting omissions, but were judged as fair [29, 30].

### Training interventions

The interventions evaluated in the review [13], four of the RCTs [16, 18, 21, 23] and one non-RCT [28] were based on established training programmes used in other settings. The remaining studies evaluated training which the authors had devised or were involved in developing. Descriptions of the training interventions and their delivery were generally insufficient for reproduction. However, details of the established programmes are available in related publications and four others online [14, 15, 20, 22]. Where details of those delivering the training were reported they generally included trainers with experience of working in mental health, such as social workers. The studies of police officers nearly all included mental health workers and police trainers, but only one also included service users as trainers [31]. Characteristics of the training interventions are given in Table 3.

**Table 3:** Characteristics of training interventions

Study	Type of training intervention	Aims of training	Target audience	Details of course content	Trainer details	Course materials
			Where delivered			
Study design	Mode of delivery		Length of training			
Police officers: Interventions with a broad mental health focus						
Compton et al. (2008) [13]	Crisis Intervention Team programs (CIT)	The CIT model is a specialised police-based program intended to enhance officers’ interactions with individuals with mental illnesses and improve the safety of all	No details or table of characteristics presented.	Not reported in this paper. Reference is made to other reports and reviews that explain the CIT model (Dupont and Cochran 2000; Cochran, Deane and Borum 2000; Munertz et al., 2006; Oliva et al., 2006; Oliva and Compton 2008)	No details or table of characteristics presented.	No details or table of characteristics presented.
					MH providers, family advocates and MH consumer groups (Cochran, Deane and Borum, 2000)	
			40 h (Cochran, Deane and Borum, 2000)			
Systematic review	Face-to-face	Parties involved in MH crises. Self-selected officers (usually volunteers selected after a review by a CIT coordinator or other senior officer) receive 40 h classroom and experiential de-escalation training in handling crises.		Cochran, Deane and Borum (2000) describe the program as teaching officers about mental illness, substance abuse, psychotropic medication, treatment modalities, patients’ rights, civil commitment law and techniques for intervening in crisis. It is also reported that ‘advocates of CIT’ state that the program promotes a philosophy of responsibility and accountability to consumers of MH services, their relatives and the community.		
	Team based					
	Mixed					
Forni et al. (2009) [29] Non-comparative study	MH awareness training: developed in house.	To improve police officers’ knowledge and awareness of MH. Objectives included: improve overall communication; understand others roles and perspectives; explore common problems and discuss ways to overcome them; understand how the Unit functions; how MH staff handle violence and aggression and for Police to understand common MH problems.	Every police officer in the borough.	Tour of unit by MH Professionals explaining their work and answering questions.	Borough police officer for MH liaison and clinicians: two police trainers responsible for teaching section 136 procedures and prosecuting offenders with MH problems	Not reported
			The Ladywell Mental Health Unit, South London and Maudsley NHS Foundation Trust, Lewisham	Assessing a mental state		
				‘Hearing voices’		
				Assaults by patients with MH problems		
	Face-to-face			Section 136 procedures		
	Team based					
	Mixed					
			One day training			
			Delivered over 4 months – groups of 12 to 20 each day			
Hansson & Markstrom (2014) [27]	An anti-stigma course added on to the regular psychiatry course as part of officer training	To improve knowledge, behaviour and attitudes towards people with mental illness	Student police officers	The programme comprised of: an introductory lecture on attitudes towards people with mental illness; a video of people with lived experience telling their story (2 h); two lectures by people with mental health problems (schizophrenia and bipolar disorder) (2 h); six videotapes of people with MH problems including psychosis, anxiety disorder, depression, bipolar disorder, suicide, and children in families with a parent with mental illness (4 h); practical in vivo training module where feedback was provided on how best to respond to specific situations (4 h). The psychiatry course consisted of 14 h of lectures on causes, diagnosis and types of mental illnesses, legislation and a case study presented at a 2 h seminar.	Two lecturers with experience of MH problems	Lectures, videos, role play with professional actors
			University			
			3 weeks full-time			
Non-RCT						
	Face-to-face					
	Team-based					
	Mixed					
Herrington & Pope (2013) [28]	Mental Health Intervention Team (MHIT)	To train uniformed officers to be specialist responders to individuals with an apparent MH concern	Front line police officers (constables, senior constables, sergeants)	Not reported in this paper. Reference is made to Crisis Intervention Teams developed in Memphis (Compton et al. 2008 and Canada et al. 2010) which MHIT is based on.	A central project team was responsible for the development and delivery of training, headed by a superintendent (commander) supported by an Education Development Officer, an analyst, and a Clinical Nurse Consultant	Not reported
Non-RCT	Not reported					
			Not reported			
			Not reported			
Norris & Cooke (2000) [30]	MH awareness training: developed in house.	To acquire awareness, understanding and skills in order to aid management of mental disorder or mental illness and thereby assist police officers in their duties.	Police officers	Morning: Legal responsibilities of police. Tour of unit by MH Professionals explaining their work and answering questions.	MH professionals at the Hutton Centre and the Cleveland police training officer	Not reported
			The Hutton Centre, Middlesborough, a medium secure unit			
				Afternoon: Assessing a mental state, emphasis on increasing awareness of signs and symptoms of MH problems rather than clinical diagnosis. Discussion of personal and professional experiences and interactive role plays and teaching.		
Non-comparative study						
	Face-to-face					
			One day training			
			Has been delivered over last five years – 132 officers trained.			
	Team based			The training emphasised the “distinction between a ‘criminal’ and a ‘mentally disordered offender’”, and the “need for care and treatment as opposed to incarceration and punishment”		
	Mixed					
Pinfold et al. (2003) [31]	Reducing psychiatric stigma and discrimination: developed in house	To communicate the ordinariness of mental ill-health and to address fear and misunderstanding surrounding experiences labelled ‘severe mental illness’. Raising participant awareness; increasing level of knowledge; changing views and affecting behaviour.	Police officers	Workshop 1: ‘What are MH problems?’ Including hearing voices simulation exercise a session on recovery and talks by an individual explaining what it feels to be psychotic.	Developed by project team, police force, Rethink severe mental illness trainers.	Workshops supported by information packs
			Two police areas in Kent			
Non-comparative study						
			2 × 2 h workshops over 6 month period.			
				Workshop 2: ‘How can the police support people with MH problems?’ including case studies and talks from carers and service users highlighting best practice principles. Additionally, workshop two reviewed mental health act 1983 legislation, local service provision and officers own MH needs	Delivered by: service users, carers, social workers, voluntary sector staff	
	Face-to-face					
	Team based					
	Mixed					
Rafacz (2012) [17]	An on-line anti-stigma program delivered in two different ways: personal experience vs information giving	The control condition aimed to increase general knowledge of specific MH conditions. The intervention condition aimed to change attitudes (reduced stigma and increased empathy)	Campus police officers	A 17 min long video in which the presenter disclosed his mental illness (schizo-affective disorder), and shared his initial experiences of the illness and treatment before discussing t where he is now and his successes, hopes and dreams for the future. He also discussed the interaction he had with police officers and their effect on the outcomes of his illness.	Videos presented by men with similar attributes to each other, however in the intervention group the presenter disclosed having a diagnosis of schizo-affective disorder	Video presentation delivered online
RCT						
			Online			
			17 min			
	Online/web-based					
	Self-directed					
	Didactic					
Police officers: Interventions with a specific mental health focus						
Bailey et al. (2001) [26]	Awareness training on intellectual disabilities	Training aimed to raise the awareness of police officers to people with intellectual disabilities in general	Trainee Police officers (post foundation training)	Involved a role-played exercise where residents from a group home on a local housing estate attended a community meeting relating to a drug problem on the estate. Police officers within the treatment group were allocated a number of roles, including that of a person with intellectual disability resident in a group home. This was followed by a debrief session and exploration of stereotyped views held about people with intellectual disability.	One of the co-authors, a professional with a background in intellectual disability services.	Briefing, role play, plenary group. Other course materials not reported
Non-RCT						
			Not stated			
	Face-to-face Team-based Interactive					
			Not stated but appeared to be a single session			
Teagardin et al. (2012) [15]	“Law Enforcement: Your Piece to the Autism Puzzle”: a video to educate law officers about Autism Spectrum Disorders (ASD)	To help the recognition and identification of and attitudes towards people with ASD.	‘In the field’ law enforcement officers.	The video covers topics included what is ASD, how to recognize persons with ASD, and how to respond to persons with ASD.	No trainer present, video only.	Video presentation
RCT			Law enforcement training venue			
			13 min			
	On-line/web based					
	Self-directed					
	Didactic					
Other non-mental health trained professionals: Interventions with a broad mental health focus						
Dorsey et al. (2012) [25]	Project Focus’ a caseworker training and consultation model. Training followed by case-specific consultation.	To enhance the skills of case-workers through training and case-based consultations to enable better recognition of MH needs and link youth with effective MH treatment (EBP). Project focus was designed to explore whether an increase in caseworker capacity to identify commonly occurring MH problems and to refer to EBPs improves child well-being.	Child Welfare Caseworkers.	The training involved: building awareness of available EBPs in the community; common MH needs for youths in foster care screening to identify needs. Three main classes of MH problems were covered: internalising (depression and anxiety), externalising (disruptive behaviour) and attention-related issues. Screening strategies were discussed. Classes of disorders rather than specific diagnosis were covered. Overviews of ‘name brand’ approaches available locally were provided; short video modelling and demonstration of EBPs; and engagement training to involve foster parents and collaborate with clinicians. Consultation sessions involved reviewing screening data and discussing the implications, treatment options and developing action plans for each case.	None stated for training. Consultation sessions were delivered by three PhD-level psychologists and one M-level social worker with 30+ years-experience in MH.	Lectures (PowerPoint), small group activity (vignette and discussion). Short modelling and video demonstrations of available EBPs
RCT			Not specified.			
			Two 3-h training sessions and following training, caseworkers received 4 months of bi-weekly 1 h case specific consultation.			
	Face-to-face					
	Team based					
	Mixed.					
Jorm et al. (2010) [23]	A modified version of the Youth MHFA course.	For teachers, to improve: knowledge and attitudes towards MH; confidence in helping students; knowledge of school policies and procedures for dealing with students with MH problems. Training also aimed to improve teachers ability to support colleagues with MH problems seek information about MH problems and their own MH	Teachers	How to apply the MHFA action plan **“ALGEE”: A**ssess the risk of suicide or harm; **L**isten non-judgementally; **G**ive reassurance and information; **E**ncourage to get appropriate professional help; **E**ncourage self-help strategies	MHFA trainers who had previously worked as teachers. Each course was taught by two instructors, one from the Department of Education and Children’s Services and the other from the Child and Adolescent Mental Health Service. Instructors got a one-week training program in how to conduct this modified Youth MHFA course. They were trained by two experienced trainers, including the person who devised the MHFA course	A lesson plan for each session, the existing Youth MHFA manual and a set of MH factsheets
			Participants’ school			
RCT						
			Two parts, taught over two days, seven hours each day			
	Face-to-face					
				Part 1 (for all education staff) covered departmental policy on MH issues, common mental disorders in adolescents and how to use the MH action plan		
	Team based					
	Mixed					
				Part 2 (for teachers with special responsibility for student welfare) provided information about first aid approaches for crises that require a more comprehensive response and about responses for less common MH problems		
		For students to improve their MH and increase information provided about MH				
Lipson et al. (2014) [21]	MHFA	For Resident Advisors and Residents trained in MHFA to have improved attitudes, knowledge and self-efficacy to manage MH issues in their residential communities. This should lead to more contact with residents about MH issues, leading to increased knowledge and attitudes at the population level. Ultimately, training aimed to increase residents’ service utilization, so improving MH.	University Resident Advisors	The five course modules were on: depression, anxiety, psychosis, substance abuse and eating disorders. Each module had information about signs and symptoms, appropriate responses, and interactive activities. The cornerstone for MHFA is a five-step gatekeeper action plan ALGEE (as above). MHFA emphasises that self-help does not replace professional care in potential crises.	Instructors were certified by the National Council on Behavioral Healthcare. Most of the instructors (10 of 14) were behavioural health clinicians.	Slides, demonstrations, and case examples.
RCT	Face-to-face		Not specified.			
	Team based					
	Interactive					
			12 h (since reduced to 8 h but still retaining breadth of content)			
Svensson & Hansson (2014) [16]	MHFA translated and modified to suit the Swedish context	To improve MH literacy among the general public and give the public the skills to be able to provide initial help to people with MH problems.	Public sector staff (social insurance agencies, employment agencies, social services, schools, police departments, correctional treatment units, rescue services, recreation centres).	The course was taught in five steps (as in ALGEE above). The steps were then applied to depression, anxiety disorders, psychosis and substance use disorder. Attendees were taught how to help a suicidal person, a person having a panic attack, a person who has experienced a traumatic event and a psychotic person threatening violence.	An Australian team taught three Swedish main instructors, who then taught 18 instructors who implemented the training program. All the instructors had experience of MH work in some form, such as health care staff and volunteers in user organisations.	An MHFA manual in Swedish
RCT						
	Face-to-face					
	Team based					
	Interactive					
			Training was given at worksites or local colleges localities in classes			
			12 h (equally spread over 2 days)			
Thombs et al. (2015) [14]	Peer Hero Training Program: uses a story-based approach to emphasise Resident Assistants (RAs) need to show courage in carrying out their responsibilities as MH & alcohol/other drug first-aid providers.	Improve skills, knowledge and confidence in managing their ‘First-aid’ response in relation to MH and alcohol/other drug situations for students living in university residences. Address the RA’s four critical attitudes which affect their ability to manage these ‘first-aid’ situations: perceived referral barriers, referral self-efficacy, referral anticipatory anxiety, and perceived referral norms.	University RAs	Program has three components, all in video format. 1) 3 dramatisations of a situation which RAs may face relating to alcohol, other drug, MH, and academic problems and methods for referring students for help. Interactive decision points require the RA to select 1 of 4 answers. The actor RA then dramatises the selected answer before feedback is given about the selected answer. 2) two-part counselling session: a student referred by an RA meeting with a counsellor, the story line flows from videos above to illustrate what students referred to counselling may encounter. 3) a series of interviews with actual parents of students and senior residence life professionals on: what is expected of RAs; not ignoring or overlooking students’ needs; how to approach students with an observed behavioural problem; need for RAs to be sincere, empathic and maintain confidentiality.	Developed by team of campus residence professional staff, RA supervisors, RAs campus MH professionals, student affairs professionals and health behaviour researchers.	Interactive videos
			Not specified			
RCT			Three separate training sessions which each took between 15 and 25 min to complete.			
					Delivered on line.	
	On-line/web-based					
	Self-directed					
	Interactive					
Other non-mental health trained professionals: Interventions with a specific mental health focus						
Hart & More (2013) [24]	Information relating to Autism Spectrum Disorder (ASD).	Improve knowledge of ASD by comparing two methods of information delivery.	Trainee teachers	The ASD-related content was based on information on the Centers for Disease Control website and the course text, included: early warning signs and current prevalence and definition of ASD. Content used reflected the local cultural and linguistic diversity and that seen in ASD: this focused on potential underservice of ethnic and linguistic minority populations and teacher strategies for developing cultural competence and collaborative relationships with families.	The content of the training was developed by the authors: no details provided. Delivery was online.	For both groups material was located on a university Blackboard Course Management Learning System which students were asked to log onto via a laptop. Those allocated to the podcast also used headphones.
			Classroom			
RCT			20 min			
	On-line/web-based					
	Self-directed					
	Didactic					
Kolko et al. (2012) [22]	Alternatives for Families: A Cognitive–Behavioral Therapy (AF-CBT)	Provide practitioners with skills to use AF-CBT when working with families with physical forces, aggression or abuse of children.	Community practitioners (clinicians)	Initial training based on the AF-CBT Session Guide: included didactic and experiential activities, case examples, group discussion, videotape reviews, and behavioural rehearsal/ challenge exercises. The session guide provides clinicians with an outline and examples for presenting the three phases of AF-CBT: engagement and psycho-education, individual skill-building, and family applications. Follow-up training: Each consultation began with a review of one or more AF-CBT topics, followed by two case presentations, feedback from consultants and the group, and problem solving to address the needs of the presenting clinicians. Booster training: Sessions focused on case conceptualisation, review of a skill topic, exploration of treatment adaptations and use of handouts, and implementation challenges.	Three experienced, ‘second generation’ trainers either alone or in tandem (1 trainer to 8–15 practitioners).	Treatment book; AF-CBT session guide; clinician-friendly handouts; two children’s books
RCT						
			Initial training: conducted at the site of each participating agency, repeated for each cohort. Follow-up: at agency site; Booster: not stated.			
	Face-to-face				Experienced AF-CBT clinicians, trainers, and developers generated the initial training content based on the AF-CBT Session Guide.	
	Team based					
	Interactive					
			Initial training: 4 × 8 h weekly workshops for 1 month; Followed by 10 × 90 mins bi-weekly group case consultations.			
			Annual booster sessions offered (1.5–2 h) from 6 to 12 months after initial training.			
McVey et al. (2008) [20]	An online programme called “The student body: promoting health at any size an online programme”	Help teachers and public health practitioners prevent the onset of disordered eating through the promotion of positive body image in children before they reach adolescence. Inform adult role models about the various factors influencing children’s body image.	Teachers and public health professionals	Six modules: media and peer pressure, healthy eating, active living teasing, adult role models and school climate. Steps in each module for the facilitator: 1) a case study introducing the topic using an animated cartoon; 2) background information providing topic information and its significance to disordered eating prevention; 3) instructions on how to conduct a classroom activity with students; 4) topic-related supplementary resources.	Online	Case study script for role-play, PDF of background information, true or false game, comic strip with game answers, parents’ handout and an optional evaluation. Topic related resources
RCT						
			Online for participants – for delivery in classroom.			
			Online-curriculum available for delivery to students during classroom time for 60 day period.			
	On-line/web-based					
	Self-directed					
	Didactic					
Moor et al. (2007) [19]	Educational package on adolescent depression	Not explicitly specified but intervention designed to help the recognition, identification of and attitudes towards adolescent depression.	Teachers	The training was delivered in three parts. 1) An introduction to adolescent depression and the importance and challenges of early detection; emphasising the role of teachers. Followed by a video of actors on ways that the signs of depression may show in school settings and standardising information on the signs and symptoms of depression. 2) Case vignettes illustrating a range of difficulties that schools may encounter were presented to teachers in small groups for discussion. All depressive disorders in the community such as co-morbidity of depression with school refusal, drug and alcohol abuse and conduct disorder were included. Management strategies appropriate for teachers to use in school were covered, including problem solving approaches. 3). Discussion of issues specific to each staff group’s local triage procedures and referral of hypothetical at-risk pupils.	Each training session was delivered by the same pair of trainers-details of trainers not provided.	An educational video and case vignettes
			Not specified			
RCT			2 h			
	Face-to-face					
	Team based					
	Mixed					
Ostberg & Rydell (2012) [18]	A modified version of Barkley’s parent training programme-adapted to setting and for use with teachers	To better equip teachers and parents with the ‘tools’ they need and “strategies in Everyday life” to help children with ADHD	Teachers and parents	Sessions from Barkley’s parent training included information about neuropsychiatric problems, teaching participants to use reinforcements, problem solving and communication with the children. Adaptations to the programme included: removal of ‘time-out’ for unwanted behaviour, home assignments were based on the problems parents and teachers experienced and reported on. Problem-solving aspect of the training was extended. A structure for co-operation between home and school was formed.	Two “well-trained” group-leaders per group	Home assignments based on problems reported by parents and teachers
			Child and adolescent psychiatry clinic.			
RCT						
			Parents 10 weekly 2 h sessions, teachers 8 sessions.			
	Face-to-face					
	Team based					
	Mixed					

Mixed = Interactive and didactic, *h* hour, *min* min

### Training for police officers: Interventions with a broad mental health focus

The systematic review focussed on CIT programmes originating in Memphis, USA. CIT is a police based response undertaken in collaboration with other services such as mental health professionals and ambulance services. These programmes provide selected officers with specialist training in dealing with mental health related calls. CIT/MHIT trained officers then provide a specialised front-line response to calls with the aim of directing those with mental health problems to treatment services rather than the judicial system.

A review of CIT programs (Compton, 2008) reported that CIT may be an effective component in connecting individuals with mental illnesses who come to the attention of police officers with appropriate psychiatric services. The limited and poor quality research identified indicates that the training component of the CIT model may have a positive effect on officers’ attitudes, beliefs, and knowledge relevant to interactions with people with mental health problems. CIT-trained officers have reported feeling better prepared in handling calls involving individuals with mental illnesses [13]. On a systems level, the review found that CIT, in comparison to other pre- and post-diversion programs, may be associated with a lower arrest rate and lower associated criminal justice costs [13]. However, the review failed to find support for the roll out of CIT.

In a non-RCT, Herrington et al. (2014) evaluated the MHIT programme in Australia [28] which is based on the CIT model. There was little change in practice or perceived quality of relationships between the police and other stakeholders [28]. There were no significant differences between the MHIT trained and non-MHIT trained officers in terms of skills, except once trained, MHIT officers reported spending less time at Mental Health Act events (trained mean 54.5mins; control 99.5 mins).

Two non-comparative studies reported on local collaborations between police forces and professionals in mental health units delivering 1 day mental health awareness training to front line police officers [29, 30]. Forni’s (2009) post training survey found a high degree of satisfaction with the training and officers said it was relevant to their daily work [29]. They also self-reported better understanding of mental health services with some myths being dispelled and terms such as ‘psychotic’ and ‘delusions’ becoming clearer. The mental health professionals who delivered the training also reported a better understanding of the role of the police and the pressures and constraints they operate under. Norris and Cooke (2000) reported a retrospective survey which aimed to establish how useful the training had been in practice [30]. Of the 55 respondents, 53 (96%) had dealt with mentally ill people, 34 (61%) had used their training and 37 (67%) felt the training had increased their ability to deal with people with mental illness. Having regular updates was felt necessary by 15 (27%) respondents.

Hansson and Markstrom’s (2014) non-RCT assessed an anti-stigma course as an addition to the regular police officer training psychiatry course [27]. Rafacz (2012) compared two ways of presenting an on-line anti-stigma programme to campus police officers in a non-RCT: personal experience versus information giving [17]. A non-comparative evaluation of an educational intervention to reduce psychiatric stigma and discrimination in the police force in England was undertaken by Pinfold et al. (2014) [31]. All three studies took a team based approach and used mixed teaching methods. Hansson and Markstrom (2014) and Pinfold (2014) delivered face-to-face training and included people with experience of mental health problems as trainers. Rafacz (2012) used two video presentations: one where the presenter disclosed his mental illness and the other with no disclosure.

Hansson and Markstrom (2014) found improved attitudes, mental health literacy and knowledge, and an increased willingness to interact with people with mental illness post intervention. Improvements were also seen at a 6 month follow-up; however this was based on data from the intervention group only [27]. In comparing anti-stigma videos of personal experience with information giving, Rafacz (2012) found neither was effective in changing attitudes [17]. The analysis suggested that attitudes of the campus police officers were generally non-stigmatising.

The non-comparative evaluation by Pinfold et al. (2003) included a pre and immediate post training satisfaction survey and a 4-week post training survey (data from 109 officers). The training aimed to reduce psychiatric stigma and discrimination [31]. A positive impact on police work post training was identified by 32 (59%) police officers, mostly through a clearer understanding leading to better communications; while 22 (41%) felt the training had made no difference to their practice. Although 77% of officers perceived an increase in their knowledge, there were no significant changes in general knowledge of mental illness and schizophrenia. Positive changes in attitude towards people with mental health problems were seen; however, the sessions did not impact on the officers’ view that people with mental health problems are likely to be violent (61% agreed at baseline, 54% at follow-up).

### Training for police officers: Interventions with a specific mental health focus

One RCT [15, 18–20, 22, 24] and one non-RCT [26] focussed on training police officers to deal with people with specific mental health conditions.

Bailey et al. (2001) undertook a non-RCT of intellectual disabilities awareness training for probationary police officers [26]. The training was team-based, interactive and delivered face-to-face at the police training centre. The control group received no specific information about people with intellectual disabilities. The pre and post training evaluation showed a statistically significant improvement in attitudes in the intervention group compared with the control group.

One RCT investigated online delivered self-directed, didactic training for police officers about autism. Teagardin et al. (2012) compared front line police officers viewing a video about autism with a control group who did not see the video [15]. Significant differences before and after training were identified for reported confidence in identifying persons with Autism Spectrum Disorder (ASD) and in interacting with persons with ASD. Intervention group knowledge of ASD also significantly improved compared with control group post training.

### Training for other non-mental health trained professionals: Interventions with a broad mental health focus

Dorsey et al. ‘s (2012) RCT examined whether a child welfare caseworker training and consultation model would improve knowledge of evidence based practice and ability to identify mental health problems and referral options [25]. Delivered face-to-face, the training was team based and included didactic and interactive elements. This was followed by 4 months of bi-weekly case specific support from a psychologist or social worker. Although the intervention group had significantly increased awareness of evidence based practice, the authors found no significant changes in practice or skills between the intervention and control groups.

The Mental Health First Aid (MHFA) programme started in Australia and has spread to other countries. Aimed originally at training adults in the general population to assist other adults, MHFA has been tailored for use by specific groups. We identified three RCTs evaluating MHFA for teachers [23], university resident advisors [21], and public sector staff such as social workers, human resource managers and employment managers [16]. All three study interventions were team based, involved interactive elements, and were delivered face-to-face, over similar timeframes (12 to 14 h over 2 days). There were no changes in practice detected for MHFA trained resident advisors, nor in take up of mental health services by the students in the care of the resident advisors [21]. However, public sector staff who received the training improved their readiness to provide help to people in mental health crisis compared with the control group, which was sustained at 2 year follow-up [16]. Additionally students of trained teachers were significantly more likely to report that they received information about mental health problems than students of un-trained teachers. Improved attitudes in the intervention group were identified in two studies [16, 23], but no effect in the third [21]. All three studies reported increases in self assessed confidence and knowledge compared with their control groups.

One cluster RCT assessed the Peer Hero Training program, a story-based approach, which was delivered in interactive video format, with self-directed learning to university resident advisors [14]. At 7 month follow-up, the resident advisors reported making more than ten times as many first-aid encounters in the past 30-days for alcohol, drug, mental health, and academic problems compared with resident advisors assigned to training-as-usual. They also reported increased confidence and skills.

### Training for other non-mental health trained professionals: Interventions with a specific mental health focus

One RCT investigated online self-directed, didactic training about autism. Hart and More’s (2013) study provided information to student teachers; the group viewing a Podcast performed statistically significantly better on the ASD comprehension test compared with the comparator group who received the same information in written format [24]. This RCT was the only included study to report an underpinning theory that was successfully applied throughout the study [24].

A face-to-face, team based, interactive intervention, “Alternative for Families: A Cognitive-Behavioral Therapy (AF-CBT)” aimed to provide practitioners with skills to use AF-CBT when working with families where there is concern about physical discipline, aggression or abuse of children. The intervention was delivered to community practitioners and outcomes compared with a control group in an RCT by Kolko et al. (2012) [22]. Intervention participants reported high levels of satisfaction with all aspects of the training, in particular the training materials. Compared with the control group at 6 month follow-up, the AF-CBT group reported a significantly greater increase for the teaching processes; knowledge about CBT; skills in dealing with a history of abuse, and general psychological skills. However, at 18 months post training, these differences were no longer significant.

McVey et al. (2008) looked at the feasibility and usefulness of an on-line modular programme to help elementary school teachers and public health practitioners prevent eating disorders [20]. The didactic module was made available to the intervention group for self-directed study any time over a 60 day period: overall a high level of satisfaction with the modules was reported by participants. Compared with the comparator group, teachers in the intervention group reported statistically significant improvements over time in their knowledge about facts concerning restrictive dieting and about peer influences. There were no significant differences between public health professionals for knowledge items; however the intervention group did demonstrate significant increases in self-efficacy to fight weight bias compared with the comparator group. Almost all (94%) of the intervention participants said the information learned would prompt them to make changes to their school environment; 74% said the program had positively influenced their own feelings about their body shape; and 93% reported that the program improved their overall delivery of body image and health eating curriculum to students.

Teachers were randomised to receive an educational package on adolescent depression or waiting list in an RCT by Moor et al. (2007) [19]. The 2 hour training was delivered face-to-face, was team based and included mixed presentation methods. Teacher responses to an attitude questionnaire showed increased confidence compared with the control group, but this did not translate into improved recognition of depressed pupils. Teachers in the experimental group recognized 52% of cases before the intervention and 45% afterwards, whilst the control teachers recognized 41% and 43%, respectively. The training produced no improvement in recognition of depressed pupils.

An RCT examined an existing parent training programme on Attention Deficit Hyperactivity Disorder (ADHD) adapted for delivery in a clinic and for use with teachers and parents [18]. Delivered face-to-face, the team based training included interactive and didactic elements. At 3 month follow up a significant reduction in parent-rated ADHD symptoms and in problematic behaviours were reported in the intervention group. For teacher ratings, significantly reduced emotional problems were found in the control group.

### Audits and evaluations carried out by police forces in England and Wales

We received 25 responses from 22 different counties after sending out 75 email requests for audits of police training in mental health. However, this did not provide further data and no audits or evaluations have to our knowledge been undertaken.

### On-going or completed but not yet published work

Our searches found a protocol for a cluster RCT on an integrated workplace mental health intervention [32]. The study is investigating whether improved leadership skills and mental health literacy leads to improved psychosocial working conditions for police officers in Southeast Australia. The ISRCTN registration lists the overall trial end date as 30/12/2016.

## Discussion

We identified evaluations of a wide variety of training interventions, populations and settings. The training programmes ranged between awareness raising, ways to change practice, and comparison of training delivery methods. The interventions also varied from addressing specific mental health conditions to providing a broad understanding of mental health illnesses and vulnerabilities, with some including how to interact effectively. There were huge variations in the design, delivery method and content of the training, and in the knowledge, experience and skills of those developing and/or delivering the training. Although 12 RCTs were identified, overall the quality of reporting makes it difficult to assess the reliability of their findings.

A number of the training interventions included dramatisations or role play, some with actors or service users. These studies found some positive effect compared with their comparison group [14, 19, 21, 22, 26, 27]. Likewise, the non-comparative studies all included role play and all reported improvement in at least one outcome [29–31]. There are a large number of reviews on adult learning practices and methods [33–36]. Dunst and Trivette (2012) reviewed 58 RCTs and found that training using a variety of presentation methods in groups of less than 40, in applied settings, over 20 h on multiple occasions was optimum for acquiring new knowledge or skills [36]. Where details were reported, most of the interventions included in this review were delivered in line with evidence based best practice.

The trainers who deliver a training package are crucial to the success of changing perceptions and behaviours. Coleman and Cotton (2014), highlight the important role of the trainer in not only delivering effective training but in the success of implementing any related programme [37].The studies of training police officers nearly all used police trainers alongside mental health professionals in delivery of interventions: potentially helping each profession to understand the others organisational culture.

A dilemma when providing training on mental health issues to non-mental health professionals is deciding what trainees need to know and in what detail. There were no studies directly comparing general versus specific training programmes. The gaps in reporting details and the wide variation in the included studies precludes drawing even tentative conclusions about general mental health awareness raising versus condition specific training programs. Over half the studies had specific mental health foci, settings and participants limiting the generalisability of the findings. For example, learning disabilities frequently co-occur with mental ill health, but many of the training interventions focussed on one specific mental health issue without consideration of the potential for other vulnerabilities. Conversely, some of the included studies used pre-existing training packages, but with little reference to previous evaluations. For example, CIT is being rolled out across the USA and elsewhere but there is little robust evidence of its effectiveness [38, 39]. Likewise, the TEMPO (Training and Education about Mental illness for Police Organizations) model for Canadian police personnel, while developed on sound principles and research evidence, has not yet had the rigorous outcome evaluation of implementation recommended by Coleman and Cotton (2014) [37]. None of the studies identified aimed to provide skills that could be used in multiple situations.

### Limitations of this review

While our searches were comprehensive it is possible that we failed to find some relevant studies. There were variations in the usefulness of tagging/thesaurus terms used in the databases searched. For example a paper in Criminal Justice Abstracts with the phrase “the impact of police training in mental health” in the title, used the thesaurus terms, “MENTAL health services/MENTAL illness/PEACE officers/POLICE reform”, with no mention of training at all. This risked the paper being missed in the combination of “police AND training”; however the paper was identified in our hand searching. The search strategies were tailored to each individual database, but there is always a balance between sensitivity and specificity. Our use of web searches, forward and backward reference searches and the request for published or unpublished audits and/or evaluations from police trainers in England and Wales may have gone someway to mitigating the risk of publication bias.

Generalisability is limited as the searches were restricted to English language and studies in OECD countries. The participants, settings and interventions were in some cases very specialist, also limiting generalisability [16, 18, 20, 24, 25].

Our research question was broad, therefore the evaluations we identified included a wide variety of training interventions, populations and settings; this could be considered a limitation. We found an overall lack of high quality evidence to inform training decisions for any non-mental health professionals coming into contact with people with a mental health issue.

Given the lack of RCTs of training specifically for police officers, we included methodologically less robust, non-randomised controlled trials: and restricted their inclusion to those in the police setting. Even within the RCTs, the heterogeneity in all aspects of the included studies made a narrative synthesis the only option, limiting the strength of the conclusions that can be drawn. While the outcome categories selected are widely used in research of training, they presented some interpretation challenges when data extracting. This was mainly because of the diversity of outcomes assessed in the included studies and a general lack of detail in the reporting of the results.

Reporting was generally incomplete, but we did not have the resources to contact authors in the hope of receiving further details.

### Implications for future research

There is a need for high quality RCTs to evaluate the impact of training programmes for non-mental health trained professionals coming into contact with people with mental health issues, and in police officers in particular. A 2012 study in a police district of 198,000 inhabitants in the Netherlands linked police data with mental health care information [40]. In 1 year the police dealt with 492 crisis situations, and in half those cases the individuals were disengaged from mental health services. The findings confirmed the important role of police officers in linking people with mental ill health to care, and the necessity for appropriate training and understanding of local mental health services and resources for front line police.

We were interested in seven well established expected outcomes of training; however many were not measured in the evaluations identified. The few studies that attempted to measure a change in outcomes for the groups of people the trainees came into contact with found little or no impact [21, 23, 28]. Obtaining good quality service user insight into the effectiveness of training is challenging, particularly when the interaction is with the police. A surrogate measure could be community satisfaction performance indicators related to mental health interactions, as recommended by The Lawrence inquiry (1999) [41]. The short follow up time was an acknowledged limitation in most of the studies. A variety of evaluation tools were used, mostly designed in-house or where a validated tool was used this was modified in some way. A recent systematic review of the measurement properties of tools measuring mental health knowledge recommends using tools with an evidence base which reach the threshold for positive ratings according to the COSMIN checklist [42].

Outcomes measured should include those important for the trainees’ roles and for the people with mental health issues. The development of a set of core outcome measures as part of and/or to inform future studies would be beneficial. The COMET initiative has a database of core outcome measures in effectiveness trials [43]. For example The Engager 2 project is developing a set of outcome measures as part of a larger project to develop and evaluate a complex intervention for prisoners with common mental health problems who are coming to the end of their sentence [44].

Future research would also benefit from more complete reporting. The Equator Network provides a single point of access to a range of useful documents [45] including the TIDierR checklist [46] and CONSORT statement [47] for the accurate reporting of interventions and RCTs respectively. Checklists and guidelines for reporting protocols and other types of studies are also available. Training interventions can include multiple elements, and be influenced by the characteristics of the setting and context in which it is being delivered and implemented. As such they may be considered complex interventions, for which specific reporting guidelines are also available [48].

## Conclusions

A variety of training programmes exist for non-mental health professionals who come into contact with people who have mental health issues. There may be some short term change in behaviour for the trainees, but longer term follow up is needed. Research evaluating training for UK police officers is needed in which a number of methodological issues need to be addressed.

## Additional files

Additional file 1: Search strategies. (DOCX 74 kb)

Additional file 2: Table of studies excluded on review of full paper. (DOCX 50 kb)

Additional file 3: Quality assessments. (DOCX 19 kb)

## Abbreviations

ACPO: Association of Chief Police Officers
ADHD: Attention deficit hyperactivity disorder
AF-CBT: Alternatives for Families: A Cognitive-Behavioural Therapy
ASD: Autism Spectrum Disorder
CIT: Crisis Intervention Team
COMET: Core Outcome Measures in Effectiveness Trials
CONSORT: Consolidated Standards of Reporting Trials
DH: Department of Health
ES: Effect size
ISRCTN: International Standard Registered Clinical/soCial sTudy Number
MHFA: Mental Health First Aid
MHIT: Mental Health Intervention Team
NPIA: National Policing Improvement Agency
OECD: Organisation for Economic Co-operation and Development
OR: Odds ratio
PROSPERO: International Prospective Register of Systematic Reviews
RCT: Randomised Controlled Trial
ROBIS: Risk of bias in systematic reviews
TIDierR: Template for Intervention Description and Replication
USA: United States of America
