# Supplementary material for: Mental health training programmes for non-mental health trained professionals coming into contact with people with mental ill health: a systematic review of effectiveness
Source: BMC Psychiatry. 2017 May 25;17:196. doi: 10.1186/s12888-017-1356-5 (PMC5445268; doi:10.1186/s12888-017-1356-5)
Supplement: Supplementary file 1 — Search strategies. (DOCX 74 kb) [file 12888_2017_1356_MOESM1_ESM.docx]

**Additional file 1: Search strategies**

**Database strategies**

**Applied Social Sciences Index and Abstracts (ASSIA) via ProQuest , search date 5^th^ November 2015. 155 records retrieved**

[(SU.EXACT("Mental health") OR (SU.EXACT("Insanity") OR SU.EXACT("Schizophrenia") OR SU.EXACT("Behaviour disorders") OR SU.EXACT.EXPLODE("Psychotic mood disorders") OR SU.EXACT("Communication disorders") OR SU.EXACT("Psychoses") OR SU.EXACT.EXPLODE("Mental illness" OR "Neuroticism" OR "Psychoticism")) OR SU.EXACT("Personality disorders") OR SU.EXACT("Bipolar affective disorder") OR SU.EXACT("Depression") OR SU.EXACT("Suicide") OR SU.EXACT("Vulnerable people") OR SU.EXACT("Learning disabilities") OR (ti("learning disabilit*" OR "learning difficult*" OR "intellectual disabilit*" OR "mental* retard*" OR "special needs") OR ab("learning disabilit*" OR "learning difficult*" OR "intellectual disabilit*" OR "mental* retard*" OR "special needs")) OR (ti("mental* ill*" OR schizophren* OR bipolar) OR ab("mental* ill*" OR schizophren* OR bipolar))) AND (((SU.EXACT("Inservice training") OR SU.EXACT("Staff development")) OR (ti(staff NEAR/3 (train* OR develop*)) OR ab(staff NEAR/3 (train* OR develop*))) OR (ti(training NEAR/3 (program* OR course* OR package* OR resource*)) OR ab(training NEAR/3 (program* OR course* OR package* OR resource*))) OR (ti(teaching NEAR/3 (program* OR course* OR package* OR resource*)) OR ab(teaching NEAR/3 (program* OR course* OR package* OR resource*))) OR (ti(educat* NEAR/3 (program* OR course* OR package* OR resource*)) OR ab(educat* NEAR/3 (program* OR course* OR package* OR resource*))) OR (ti(learning NEAR/3 (program* OR course* OR package* OR resource*)) OR ab(learning* NEAR/3 (program* OR course* OR package* OR resource*))) OR (ti((internet OR web*) NEAR/3 (program* OR course* OR package* OR resource*)) OR ab((internet OR web*) NEAR/3 (program* OR course* OR package* OR resource*))) OR (ti(skill* NEAR/3 (program* OR course* OR package* OR resource* OR develop*)) OR ab(skill* NEAR/3 (program* OR course* OR package* OR resource* OR develop*))) OR SU.EXACT("Computer assisted training")) AND ((SU.EXACT.EXPLODE("Community policing" OR "Law enforcement" OR "Policing" OR "Sector policing" OR "Transnational policing") OR SU.EXACT.EXPLODE("Chief constables" OR "Community police officers" OR "Custody officers" OR "Detectives" OR "Former police officers" OR "Liaison police officers" OR "Metropolitan commissioners" OR "Mounted police officers" OR "Police inspectors" OR "Police officers" OR "Police superintendents" OR "Private investigators" OR "Witness liaison officers")) OR (SU.EXACT("Juvenile justice") OR SU.EXACT("Criminal justice")) OR SU.EXACT.EXPLODE("Maximum security prisons" OR "Prisons" OR "Remand prisons" OR "Secure units") OR (ti((police OR prison* OR jail* OR gaol*) NEAR/2 (personnel OR staff OR worker* OR caseworker* OR office* OR official* OR employee* OR warder*)) OR ab((police OR prison* OR jail* OR gaol*) NEAR/2 (personnel OR staff OR worker* OR caseworker* OR office* OR official* OR employee* OR warder*))) OR (ti((probation OR parole OR court* OR justice) NEAR/2 (personnel OR staff OR worker* OR caseworker* OR office* OR official* OR employee* OR warder*)) OR ab((probation OR parole OR court* OR justice) NEAR/2 (personnel OR staff OR worker* OR caseworker* OR office* OR official* OR employee* OR warder*))) OR (ti(magistrate* OR para-medic* OR paramedic*) OR ab((magistrate* or para-medic* OR paramedic*))) OR (ab((emergency OR ambulance OR casualty) NEAR/2 (personnel OR staff OR worker* OR office* OR official* OR employee*)) OR ti((emergency OR ambulance OR casualty) NEAR/2 (personnel OR staff OR worker* OR office* OR official* OR employee*))) OR (ti((charity OR voluntary) NEAR/2 (personnel OR staff OR worker* OR office* OR official* OR employee*)) OR ab((charity OR voluntary) NEAR/2 (personnel OR staff OR worker* OR office* OR official* OR employee*))) OR (SU.EXACT("Victims") OR ti(crime NEAR/2 (witness* OR victim* OR suspect*)) OR ab(crime NEAR/2 (witness* OR victim* OR suspect*)) OR ti(teacher* OR schoolteacher* OR instructor* OR lecturer*) OR ab(teacher* OR schoolteacher* OR instructor* OR lecturer*) OR ti("appropriate adult*") OR ab("appropriate adult*"))))](http://search.proquest.com/myresearch/savedsearches.checkdbssearchlink:rerunsearch/951431/SavedSearches?site=assia&t:ac=SavedSearches)

**Cochrane Central Register of Controlled Clinical Trials (CENTRAL) searched 3^rd^ November 2015 via the Cochrane Library. 143 records retrieved**

**Search Name: Police Reviews Training**

**Last Saved: 03/11/2015 11:42:49.992**

**Description: 3rd November 2015**

#1 MeSH descriptor: [Law Enforcement] explode all trees

#2 MeSH descriptor: [Jurisprudence] explode all trees

#3 criminal justice:ti,ab,kw or youth justice:ti,ab,kw or juvenile justice:ti,ab,kw or "law enforcement":ti,ab,kw (Word variations have been searched)

#4 MeSH descriptor: [Police] explode all trees

#5 MeSH descriptor: [Prisons] explode all trees

#6 ((police or prison* or jail* or gaol*) near/2 (personnel or staff or worker* or caseworker* or office* or official* or employee* or warder*)):ti,ab,kw (Word variations have been searched)

#7 ((probation or parole or court* or justice) near/2 (personnel or staff or worker* or caseworker* or office* or official* or employee*)):ti,ab,kw (Word variations have been searched)

#8 magistrate* or volunteer*:ti,ab,kw (Word variations have been searched)

#9 MeSH descriptor: [Emergency Medical Technicians] explode all trees

#10 paramedic* or para-medic*:ti,ab,kw (Word variations have been searched)

#11 ((emergency or ambulance or casualty) near/2 (personnel or staff or worker* or office* or official* or employee*)):ti,ab,kw (Word variations have been searched)

#12 ((charity or voluntary) near/2 (personnel or staff or worker* or office* or official* or employee*)):ti,ab,kw (Word variations have been searched)

#13 MeSH descriptor: [Crime Victims] explode all trees

#14 (crime near/4 (witness* or victim* or suspect*)):ti,ab,kw (Word variations have been searched)

#15 teacher* or schoolteacher* or instructor* or lecturer*:ti,ab,kw (Word variations have been searched)

#16 appropriate adult*:ti,ab,kw (Word variations have been searched)

#17 #1 or #2 or #3 or #4 or #5 or #6 or #7 or #8 or #9 or #10 or #11 or #12 or #13 or #14 or #15 or #16

#18 MeSH descriptor: [Inservice Training] explode all trees

#19 MeSH descriptor: [Staff Development] explode all trees

#20 ((training) near/3 (program* or course* or package* or resource*)):ti,ab,kw or ((teaching) near/3 (program* or course* or package* or resource*)):ti,ab,kw or ((educat*) near/3 (program* or course* or package* or resource*)):ti,ab,kw or ((learning) near/3 (program* or course* or package* or resource*)):ti,ab,kw or ((internet or web*) near/2 (program* or course* or package* or resource* or train* or educat* or learn*)):ti,ab,kw (Word variations have been searched)

#21 MeSH descriptor: [Computer-Assisted Instruction] explode all trees

#22 #18 or #19 or #20 or #21

#23 MeSH descriptor: [Mental Health] explode all trees

#24 MeSH descriptor: [Mental Disorders] explode all trees

#25 MeSH descriptor: [Schizophrenia] explode all trees

#26 MeSH descriptor: [Personality Disorders] explode all trees

#27 MeSH descriptor: [Bipolar Disorder] explode all trees

#28 MeSH descriptor: [Depressive Disorder] explode all trees

#29 MeSH descriptor: [Depression] explode all trees

#30 MeSH descriptor: [Vulnerable Populations] explode all trees

#31 MeSH descriptor: [Social Stigma] explode all trees

#32 MeSH descriptor: [Learning Disorders] explode all trees

#33 "learning disabilit*":ti,ab,kw or "leaning difficult*":ti,ab,kw or "intellectual disabilit*":ti,ab,kw or "mental* retard*":ti,ab,kw or "special needs":ti,ab,kw (Word variations have been searched)

#34 "mental* ill*" or schizophren* or bipolar:ti,ab,kw (Word variations have been searched)

#35 #23 or #24 or #25 or #26 or #27 or #28 or #29 or #30 or #31 or #32 or #33 or #34

#36 #17 and #22 and #35

#37 MeSH descriptor: [Mentally Disabled Persons] explode all trees

#38 MeSH descriptor: [Mentally Ill Persons] explode all trees

#39 #37 or #38

#40 MeSH descriptor: [Police] explode all trees

#41 #39 and #40

#42 MeSH descriptor: [Police] explode all trees and with qualifier(s): [Education - ED]

#43 #36 or #42

**Criminal Justice Abstracts searched 2^nd^ November 2015 via EBSCO. 2497 records retrieved**

| **Search Terms** | **Search Options** |
| --- | --- |
| S26 | S20 OR S24  **Limiters** - Publication Date: 19950101-20151231; Language: English |
| S25 | S20 OR S24 |
| S24 | S7 AND S23 |
| S23 | S21 OR S22 |
| S22 | TX court* N2 (mental* or schizophren* or vulnerab* or disabilit* or depress*) |
| S21 | TX policing N2 (mental* or schizophren* or vulnerab* or disabilit* or depress*) |
| S20 | S7 AND S12 AND S19 |
| S19 | S12 OR S13 OR S14 OR S15 OR S16 OR S17 OR S18 |
| S18 | TX (victim* or witness* or suspect*) |
| S17 | TX appropriate adult* OR TX ( teacher* or tutor* or lecturer* or instructor* or professor* ) |
| S16 | TX ( paramedic or ems or emergency medical service or emergency room* or prehospital ) OR TX ( ambulance N2 (personnel or staff) ) OR TX ( casualty N2 (personnel or staff) ) |
| S15 | TX youth justice OR TX juvenile justice |
| S14 | TX police OR TX ( prison* or jail* or gaol* ) OR TX ( probation or parole or court* or magistrate* or judge* ) |
| S13 | TX law enforcement OR TX correction* OR TX criminal justice |
| S12 | S8 OR S9 OR S10 OR S11 |
| S11 | TX "learning disabilit*" OR TX "intellectual disabilit*" OR TX "mental retard*" OR TX "special need*" OR TX "learning difficult*" |
| S10 | TX vulnerability OR TX vulnerable OR TX homeless |
| S9 | TX schizophren* OR TX bipolar OR TX personality disorder OR TX depressi* |
| S8 | TX "mental health" OR TX "mental disorder*" OR TX "mental* ill*" |
| S7 | S1 OR S2 OR S3 OR S4 OR S5 OR S6 |
| S6 | skills N2 (program* or course* or package* or resource* or develop*) |
| S5 | learning N2 (program* or course* or package* or resource*) |
| S4 | educat* N2 (program* or course* or package* or resource*) |
| S3 | training N2 (program* or course* or package* or resource*) |
| S2 | SU training |
| S1 | SU education |

**Embase via OVID. Searched 2^nd^ November 2015. 1247 records retrieved**

**Database: Embase <1980 to 2015 Week 44>**

**Search Strategy:**

1 law enforcement/ or jurisprudence/ or criminal justice/ (43071)

2 (criminal justice or youth justice or juvenile justice or law enforcement).ti,ab. (7388)

3 police/ or prisons/ or probation/ (20157)

4 ((police or prison$ or jail$ or gaol$) adj2 (personnel or staff or worker$ or caseworker$ or office$ or official$ or employee$ or warder$)).ti,ab. (2724)

5 ((probation or parole or court$ or justice) adj2 (personnel or staff or worker$ or caseworker$ or office$ or official$ or employee$)).ti,ab. (493)

6 (magistrate$ or volunteer$).ti,ab. (194347)

7 Rescue Personnel/ (6216)

8 (paramedic$ or para-medic$).ti,ab. (8023)

9 ((emergency or ambulance or casualty) adj2 (personnel or staff or worker$ or office$ or official$ or employee$)).ti,ab. (3424)

10 ((charity or voluntary) adj2 (personnel or staff or worker$ or office$ or official$ or employee$)).ti,ab. (262)

11 crime victim/ (959)

12 (crime adj4 (witness$ or victim$ or suspect$)).ti,ab. (965)

13 Teacher/ (23928)

14 (teacher$ or schoolteacher$ or instructor$ or lecturer$).ti,ab. (46052)

15 appropriate adult$.ti,ab. (93)

16 1 or 2 or 3 or 4 or 5 or 6 or 7 or 8 or 9 or 10 or 11 or 12 or 13 or 14 or 15 (334443)

17 In Service training/ or Professional Development/ (20181)

18 (staff adj2 (train$ or develop$)).ti,ab. (10718)

19 (training adj3 (program$ or course$ or package$ or resource$)).ti,ab. (49485)

20 (teaching adj3 (program$ or course$ or package$ or resource$)).ti,ab. (6244)

21 (educat$ adj3 (program$ or course$ or package$ or resource$)).ti,ab. (57549)

22 (learning adj3 (program$ or course$ or package$ or resource$)).ti,ab. (5729)

23 ((internet or web$) adj2 (program$ or course$ or package$ or resource$ or train$ or educat$ or learn$)).ti,ab. (6922)

24 (skill$ adj3 (program$ or course$ or package$ or resource$ or develop$)).ti,ab. (16210)

25 17 or 18 or 19 or 20 or 21 or 22 or 23 or 24 (156351)

26 Mental Health/ (86167)

27 exp Mental Disease/ (1650243)

28 exp Schizophrenia/ (149893)

29 exp Personality Disorder/ (49454)

30 exp Bipolar Disorder/ (46054)

31 exp Depression/ (343768)

32 Suicide/ (44074)

33 Vulnerable Population/ (8853)

34 Social Stigma/ (3064)

35 exp Learning Disorder/ (27484)

36 (learning disabilit$ or learning difficult$ or intellectual disabilit$ or mental retard$ or special need$).ti,ab. (54195)

37 (mental$ ill$ or schizophren$ or bipolar).ti,ab. (206567)

38 26 or 27 or 28 or 29 or 30 or 31 or 32 or 33 or 34 or 35 or 36 or 37 (1752275)

39 16 and 25 and 38 (1430)

40 mentally disabled persons/ or mentally ill persons/ (20113)

41 Police/ (8402)

42 40 and 41 (111)

43 39 or 42 (1533)

44 limit 43 to (english language and yr="1995 -Current") (1247)

**ERIC via EBSCO. Searched 6^th^ November 2015. 2915 records retrieved.**

| [Search ID#](javascript:__doPostBack('ctl00$ctl00$MainContentArea$MainContentArea$historyControl$ReorderHistoryLink','')) | **Search Terms** |
| --- | --- |
| S28 | S14 AND S20 AND S27 |
| S27 | S21 OR S22 OR S23 OR S24 OR S25 OR S26 |
| S26 | TX mental* ill* |
| S25 | TX social stigma OR TX learning disabilit* OR TX learning difficult* OR TX intellectual disabilit* OR TX mental retard* OR TX special needs |
| S24 | TX personality disorder* OR TX bipolar disoder* |
| S23 | (((ZU "schizophrenia")) or ((ZU "depression (psychology)"))) or ((ZU "suicide")) |
| S22 | (ZU "mental disorders") |
| S21 | (ZU "mental health") or (ZU "mental illness") or (ZU "mental retardation") |
| S20 | S15 OR S16 OR S17 or S18 or s19 |
| S19 | (ZU "computer assisted instruction") |
| S18 | skill* N2 (program* or course* or package* or resource* or develop*) |
| S17 | TX ( (training N2 (program* or course* or package* or resource*)) ) OR TX ( (teaching N2 (program* or course* or package* or resource*)) ) OR TX ( (educat* N2 (program* or course* or package* or resource*)) ) OR TX ( (learning N2 (program* or course* or package* or resource*)) ) OR TX ( ((internet or web) N2 (program* or course* or package* or resource* or train* or educat* or learn*)) ) |
| S16 | TX (staff train*) or (staff develop*) |
| S15 | (((ZU "training")) or ((ZU "staff development"))) or ((ZU "professional development")) |
| S14 | S1 OR S2 OR S3 OR S4 OR S5 OR S6 OR S7 OR S8 OR S9 OR S10 OR S11 OR S12 OR S13 |
| S13 | TX appropriate adult* |
| S12 | teacher* or schoolteacher* or instructor* or lecturer* |
| S11 | (crime N2 (witness* or victim* or suspect*)) |
| S10 | (ZU "victims of crime") |
| S9 | TX ((charity or voluntary) N2 (personnel or staff or worker* or office* or official* or employee*)) |
| S8 | TX ((emergency or ambulance or casualty) N2 (personnel or staff or worker* or office* or official* or employee*)) |
| S7 | TX paramedic* or para-medic* |
| S6 | TX magistrate* |
| S5 | TX ((probation or parole or court*) N2 (personnel or staff or worker* or caseworker* or office* or official* or employee*)) |
| S4 | TX ((police or prison* or jail* or gaol*) N2 (personnel or staff or worker* or caseworker* or office* or official* or employee* or warder*)) |
| S3 | (ZU "police") |
| S2 | TX criminal justice OR TX youth justice OR TX juvenile justice OR law enforcement |
| S1 | (ZU "law enforcement") |

**MEDLINE via OVID. Searched 2^nd^ November 2015. 907 records retrieved**

**Database: Ovid MEDLINE(R) In-Process & Other Non-Indexed Citations and Ovid MEDLINE(R) <1946 to Present>**

**Search Strategy:**

1 law enforcement/ or jurisprudence/ (32027)

2 (criminal justice or youth justice or juvenile justice or law enforcement).ti,ab. (6038)

3 police/ or prisons/ (11402)

4 ((police or prison$ or jail$ or gaol$) adj2 (personnel or staff or worker$ or caseworker$ or office$ or official$ or employee$ or warder$)).ti,ab. (2227)

5 ((probation or parole or court$ or justice) adj2 (personnel or staff or worker$ or caseworker$ or office$ or official$ or employee$)).ti,ab. (417)

6 (magistrate$ or volunteer$).ti,ab. (159640)

7 Emergency Medical Technicians/ (5178)

8 (paramedic$ or para-medic$).ti,ab. (6142)

9 ((emergency or ambulance or casualty) adj2 (personnel or staff or worker$ or office$ or official$ or employee$)).ti,ab. (2669)

10 ((charity or voluntary) adj2 (personnel or staff or worker$ or office$ or official$ or employee$)).ti,ab. (236)

11 crime victims/ (6119)

12 (crime adj4 (witness$ or victim$ or suspect$)).ti,ab. (729)

13 (teacher$ or schoolteacher$ or instructor$ or lecturer$).ti,ab. (39432)

14 appropriate adult$.ti,ab. (72)

15 1 or 2 or 3 or 4 or 5 or 6 or 7 or 8 or 9 or 10 or 11 or 12 or 13 or 14 (265012)

16 exp inservice training/ or staff development/ (25343)

17 (staff adj2 (train$ or develop$)).ti,ab. (7787)

18 (training adj3 (program$ or course$ or package$ or resource$)).ti,ab. (38740)

19 (teaching adj3 (program$ or course$ or package$ or resource$)).ti,ab. (5005)

20 (educat$ adj3 (program$ or course$ or package$ or resource$)).ti,ab. (46112)

21 (learning adj3 (program$ or course$ or package$ or resource$)).ti,ab. (4380)

22 ((internet or web$) adj2 (program$ or course$ or package$ or resource$ or train$ or educat$ or learn$)).ti,ab. (5245)

23 (skill$ adj3 (program$ or course$ or package$ or resource$ or develop$)).ti,ab. (12589)

24 Computer-Assisted Instruction/ (10110)

25 16 or 17 or 18 or 19 or 20 or 21 or 22 or 23 or 23 or 24 (138192)

26 Mental Health/ (24356)

27 exp Mental Disorders/ (1023375)

28 exp Schizophrenia/ (90530)

29 exp Personality Disorders/ (33215)

30 exp Bipolar Disorder/ (34179)

31 exp Depressive Disorder/ (87313)

32 Depression/ (85986)

33 Suicide/ (33043)

34 Vulnerable Populations/ (6784)

35 Social Stigma/ (2695)

36 exp Learning Disorders/ (19926)

37 (learning disabilit$ or learning difficult$ or intellectual disabilit$ or mental retard$ or special need$).ti,ab. (43042)

38 (mental$ ill$ or schizophren$ or bipolar).ti,ab. (162664)

39 26 or 27 or 28 or 29 or 30 or 31 or 32 or 33 or 34 or 35 or 36 or 37 or 38 (1184195)

40 15 and 25 and 39 (1005)

41 mentally disabled persons/ or mentally ill persons/ (6608)

42 Police/ (3702)

43 41 and 42 (48)

44 Police/ed [Education] (235)

45 40 or 43 or 44 (1239)

46 limit 45 to (english language and yr="1995 -Current") (907)

**PsycINFO via OVID. Searched 2^nd^ November 2015. 1674 records retrieved**

**Database: PsycINFO <1967 to October Week 4 2015>**

**Search Strategy:**

1 law enforcement/ or exp criminal justice/ (11806)

2 (criminal justice or youth justice or juvenile justice or law enforcement).ti,ab. (15253)

3 police personnel/ or prisons/ (12659)

4 ((police or prison$ or jail$ or gaol$) adj2 (personnel or staff or worker$ or caseworker$ or office$ or official$ or employee$ or warder$)).ti,ab. (5255)

5 Probation/ or Parole/ (1653)

6 ((probation or parole or court$ or justice) adj2 (personnel or staff or worker$ or caseworker$ or office$ or official$ or employee$)).ti,ab. (1746)

7 (magistrate$ or volunteer$).ti,ab. (30706)

8 (paramedic$ or para-medic$).ti,ab. (641)

9 ((emergency or ambulance or casualty) adj2 (personnel or staff or worker$ or office$ or official$ or employee$)).ti,ab. (749)

10 ((charity or voluntary) adj2 (personnel or staff or worker$ or office$ or official$ or employee$)).ti,ab. (214)

11 crime victims/ (3870)

12 (crime adj4 (witness$ or victim$ or suspect$)).ti,ab. (2219)

13 exp Teachers/ (58257)

14 (teacher$ or schoolteacher$ or instructor$ or lecturer$).ti,ab. (146165)

15 appropriate adult$.ti,ab. (67)

16 1 or 2 or 3 or 4 or 5 or 6 or 7 or 8 or 9 or 10 or 11 or 12 or 13 or 14 or 15 (222862)

17 Inservice training/ or professional development/ (14367)

18 (staff adj2 (train$ or develop$)).ti,ab. (5307)

19 (training adj3 (program$ or course$ or package$ or resource$)).ti,ab. (28243)

20 (teaching adj3 (program$ or course$ or package$ or resource$)).ti,ab. (4735)

21 (educat$ adj3 (program$ or course$ or package$ or resource$)).ti,ab. (35504)

22 (learning adj3 (program$ or course$ or package$ or resource$)).ti,ab. (7577)

23 ((internet or web$) adj2 (program$ or course$ or package$ or resource$ or train$ or educat$ or learn$)).ti,ab. (3699)

24 (skill$ adj3 (program$ or course$ or package$ or resource$ or develop$)).ti,ab. (20015)

25 Computer-Assisted Instruction/ (14215)

26 17 or 18 or 19 or 20 or 21 or 22 or 23 or 24 or 25 (117465)

27 Mental Health/ (44818)

28 exp Mental Disorders/ (474285)

29 exp Schizophrenia/ (75079)

30 exp Personality Disorders/ (22528)

31 exp Bipolar Disorder/ (21532)

32 exp Major Depression/ (102770)

33 Suicide/ (21590)

34 At Risk Populations/ (32662)

35 Stigma/ (7588)

36 exp Learning Disorders/ (30667)

37 (learning disabilit$ or learning difficult$ or intellectual disabilit$ or mental retard$ or special need$).ti,ab. (44310)

38 (mental$ ill$ or schizophren$ or bipolar).ti,ab. (148867)

39 27 or 28 or 29 or 30 or 31 or 32 or 33 or 34 or 35 or 36 or 37 or 38 (629505)

40 16 and 26 and 39 (2175)

41 limit 40 to yr="1995 -Current" (1674)

**Social Science Citation Index via Web of Science.**

**Searched 6^th^ November 2015. 745 records retrieved**

| **Set** | **Results** | **[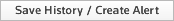](javascript:sss_overlay();) [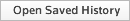](http://apps.webofknowledge.com/OutboundService.do?product=WOS&action=go&SID=Z2UX8UFU2rRla5CX2Ag&mode=sssHome&component=sss)** | **Edit Sets** | **Combine Sets**  ** AND   OR**  **** | **Delete Sets**  **  ** |
| --- | --- | --- | --- | --- | --- |
|  | | | | | |
| # 9 | [**745**](http://apps.webofknowledge.com/summary.do?product=WOS&doc=1&qid=11&SID=Z2UX8UFU2rRla5CX2Ag&search_mode=CombineSearches&update_back2search_link_param=yes) | #8 AND #7 AND #6  *Indexes=SSCI Timespan=1995-2015* | [Edit](http://apps.webofknowledge.com/WOS_AdvancedSearch_input.do?product=WOS&SID=Z2UX8UFU2rRla5CX2Ag&search_mode=AdvancedSearch&replaceSetId=9&editState=init) | Select to combine sets. | Select to delete this set. |
|  | | | | | |
| # 8 | [**350,459**](http://apps.webofknowledge.com/summary.do?product=WOS&doc=1&qid=10&SID=Z2UX8UFU2rRla5CX2Ag&search_mode=GeneralSearch&update_back2search_link_param=yes) | **TOPIC:** ("mental health" or "mental disorder*") *OR* **TOPIC:** (schizophren*) *OR* **TOPIC:** ("personality disorder") *OR* **TOPIC:** ("bipolar disorder") *OR***TOPIC:** ("depressive disorder*" OR depression) *OR* **TOPIC:** (suicide) *OR* **TOPIC:** ("vulnerable population*") *OR* **TOPIC:** ("social stigma") *OR* **TOPIC:**("learning disabilit*" or "learning difficult*" or "intellectual disabilit*" or "mental retard*" or "special needs") *OR* **TOPIC:** ("mental* ill*")  *Indexes=SSCI Timespan=1995-2015* | [Edit](http://apps.webofknowledge.com/WOS_AdvancedSearch_input.do?product=WOS&SID=Z2UX8UFU2rRla5CX2Ag&search_mode=AdvancedSearch&replaceSetId=8&editState=init) | Select to combine sets. | Select to delete this set. |
|  | | | | | |
| # 7 | [**64,688**](http://apps.webofknowledge.com/summary.do?product=WOS&doc=1&qid=9&SID=Z2UX8UFU2rRla5CX2Ag&search_mode=GeneralSearch&update_back2search_link_param=yes) | **TOPIC:** ("inservice training") *OR* **TOPIC:** ("staff development") *OR* **TOPIC:** (staff NEAR/2 (train* or develop*)) *OR* **TOPIC:** (training NEAR/2 (program* or course* or package* or resource*)) *OR* **TOPIC:** (teaching NEAR/2 (program* or course* or package* or resource*)) *OR* **TOPIC:** (educat* NEAR/2 (program* or course* or package* or resource*)) *OR* **TOPIC:** (learning NEAR/2 (program* or course* or package* or resource*)) *OR* **TOPIC:** ((internet or web*) NEAR/2 (program* or course* or package* or resource* or train* or educat* or learn*)) *OR* **TOPIC:** (skill* NEAR/2 (program* or course* or package* or resource* or develop*)) *OR* **TOPIC:** (computer NEAR/2 instruction)  *Indexes=SSCI Timespan=1995-2015* | [Edit](http://apps.webofknowledge.com/WOS_AdvancedSearch_input.do?product=WOS&SID=Z2UX8UFU2rRla5CX2Ag&search_mode=AdvancedSearch&replaceSetId=7&editState=init) | Select to combine sets. | Select to delete this set. |
|  | | | | | |
| # 6 | [**110,087**](http://apps.webofknowledge.com/summary.do?product=WOS&doc=1&qid=8&SID=Z2UX8UFU2rRla5CX2Ag&search_mode=CombineSearches&update_back2search_link_param=yes) | #5 OR #4 OR #3 OR #2 OR #1  *Indexes=SSCI Timespan=1995-2015* | [Edit](http://apps.webofknowledge.com/WOS_AdvancedSearch_input.do?product=WOS&SID=Z2UX8UFU2rRla5CX2Ag&search_mode=AdvancedSearch&replaceSetId=6&editState=init) | Select to combine sets. | Select to delete this set. |
|  | | | | | |
| # 5 | [**71,953**](http://apps.webofknowledge.com/summary.do?product=WOS&doc=1&qid=7&SID=Z2UX8UFU2rRla5CX2Ag&search_mode=AdvancedSearch&update_back2search_link_param=yes) | TS=(teacher* OR schoolteacher* OR instructor* OR lecturer*)  *Indexes=SSCI Timespan=1995-2015* | [Edit](http://apps.webofknowledge.com/WOS_AdvancedSearch_input.do?product=WOS&SID=Z2UX8UFU2rRla5CX2Ag&search_mode=AdvancedSearch&replaceSetId=5&editState=init) | Select to combine sets. | Select to delete this set. |
|  | | | | | |
| # 4 | [**3,864**](http://apps.webofknowledge.com/summary.do?product=WOS&doc=1&qid=5&SID=Z2UX8UFU2rRla5CX2Ag&search_mode=GeneralSearch&update_back2search_link_param=yes) | **TOPIC:** ((emergency or ambulance or casualty) NEAR/2 (personnel or staff or worker* or office* or official* or employee*)) *OR* **TOPIC:** ((charity or voluntary) NEAR/2 (personnel or staff or worker* or office* or official* or employee*)) *OR* **TOPIC:** (crime NEAR/4 (witness* or victim* or suspect*)) *OR***TOPIC:** ("appropriate adult*")  *Indexes=SSCI Timespan=1995-2015* | [Edit](http://apps.webofknowledge.com/WOS_AdvancedSearch_input.do?product=WOS&SID=Z2UX8UFU2rRla5CX2Ag&search_mode=AdvancedSearch&replaceSetId=4&editState=init) | Select to combine sets. | Select to delete this set. |
|  | | | | | |
| # 3 | [**19,921**](http://apps.webofknowledge.com/summary.do?product=WOS&doc=1&qid=4&SID=Z2UX8UFU2rRla5CX2Ag&search_mode=GeneralSearch&update_back2search_link_param=yes) | **TOPIC:** (magistrate* or volunteer*) *OR* **TOPIC:** (paramedic*)  *Indexes=SSCI Timespan=1995-2015* | [Edit](http://apps.webofknowledge.com/WOS_AdvancedSearch_input.do?product=WOS&SID=Z2UX8UFU2rRla5CX2Ag&search_mode=AdvancedSearch&replaceSetId=3&editState=init) | Select to combine sets. | Select to delete this set. |
|  | | | | | |
| # 2 | [**4,880**](http://apps.webofknowledge.com/summary.do?product=WOS&doc=1&qid=3&SID=Z2UX8UFU2rRla5CX2Ag&search_mode=GeneralSearch&update_back2search_link_param=yes) | **TOPIC:** ((police or prison* or jail* or gaol*) NEAR/2 (personnel or staff or worker* or caseworker* or office* or official* or employee* or warder*)) *OR***TOPIC:** ((probation or parole or court* or justice) NEAR/2 (personnel or staff or worker* or caseworker* or office* or official* or employee* or warder*))  *Indexes=SSCI Timespan=1995-2015* | [Edit](http://apps.webofknowledge.com/WOS_AdvancedSearch_input.do?product=WOS&SID=Z2UX8UFU2rRla5CX2Ag&search_mode=AdvancedSearch&replaceSetId=2&editState=init) | Select to combine sets. | Select to delete this set. |
|  | | | | | |
| # 1 | [**11,641**](http://apps.webofknowledge.com/summary.do?product=WOS&doc=1&qid=6&SID=Z2UX8UFU2rRla5CX2Ag&search_mode=AdvancedSearch&update_back2search_link_param=yes) | TS=("criminal justice") OR TS=("youth justice") OR TS=("juvenile justice") OR TS=("law enforcement")  *Indexes=SSCI Timespan=1995-2015* | [Edit](http://apps.webofknowledge.com/WOS_AdvancedSearch_input.do?product=WOS&SID=Z2UX8UFU2rRla5CX2Ag&search_mode=AdvancedSearch&replaceSetId=1&editState=init) | Select to combine sets. | Select to delete this set. |
|  | | | | | |
|  |  |  |  |  AND  OR  **** | **  ** |

**Mental health websites searched**

The websites of the following organisations were searched on 10.12.2015

- NHS Confederation’s Mental Health Network: <http://www.nhsconfed.org/resources/2014/02/mental-health-and-crisis-care>
- Mental Health and policing: improving crisis care
- Sainsbury’s centre for mental health now Centre for Mental Health): <http://www.centreformentalhealth.org.uk/personality-disorder-and-complex-needs>
- [[1-3](#_ENREF_1)]
- The Bradley Commission: Personality disorder and complex needs
- The police foundation: <http://www.police-foundation.org.uk/uploads/holding/policy/policing_and_mental_health_resp.pdf>
- [Mental Health Foundation](http://www.mentalhealth.org.uk/)
  [Together](http://www.together-uk.org/) for mental wellbeing
  [The Centre for Mental Health](http://www.scmh.org.uk/)
- [Depression Alliance](http://www.depressionalliance.org/)
- Black Mental Health
- [British Association for Counselling and Psychotherapy](http://www.bacp.co.uk/)
- Mind: AH-M Spoke with them. The training they offer (and evaluate) focusses on emergency service personnel and their mental health and resilience, rather than those that they come into contact with.
- Rethink
- [Breakthrough](http://www.breakthroughmhart.com/)
- [PANDAS Foundation](http://www.pandasfoundation.org.uk/)
- [Young Minds](http://www.youngminds.org.uk/)
  020 7336 8445
  Provides information and advice for anyone with concerns about the mental health of a child or young person.
- The [Very Important Kids](http://www.vik.org.uk/) website has been created by YoungMinds for young people affected by mental health problems. Here you can get advice, share experiences and feed into the work they do.
- [Childline](http://www.childline.org.uk/)
- [Nightline](http://nightline.ac.uk/)
- [Age Concern](http://www.ageuk.org.uk/)
  [Refugee Council](http://www.refugeecouncil.org.uk/)
- [Anxiety UK](https://www.anxietyuk.org.uk/)
  08444 775 774
  Works to relieve and support those living with anxiety disorders by providing information, support and understanding via an extensive range of services, including 1:1 therapy.
- SANE
- CALM
- Scottish association for mental health

**Scanning for relevant ongoing work and work completed but not yet published**

NHS England website ([www.england.nhs.uk](http://www.england.nhs.uk))

DOH website ([www.gov.uk/government/organisations/department-of-health](http://www.gov.uk/government/organisations/department-of-health))

Clinicaltrials.gov ([www.clinicaltrials.gov](http://www.clinicaltrials.gov) )

ISRCTN register ([www.isrctn.com](http://www.isrctn.com))

PROSPERO ([www.crd.york.ac.uk/PROSPERO](http://www.crd.york.ac.uk/PROSPERO))
