# Supplementary material for: Mental health training programmes for non-mental health trained professionals coming into contact with people with mental ill health: a systematic review of effectiveness
Source: BMC Psychiatry. 2017 May 25;17:196. doi: 10.1186/s12888-017-1356-5 (PMC5445268; doi:10.1186/s12888-017-1356-5)
Supplement: Supplementary file 2 — Table of studies excluded on review of full paper. (DOCX 50 kb) [file 12888_2017_1356_MOESM2_ESM.docx]

**Additional file 2. Table of studies excluded on review of full paper**

| **Reference** | **Reason for exclusion** |
| --- | --- |
| Cox AD, Dube C, Temple B. The influence of staff training on challenging behaviour in individuals with intellectual disability: a review. Journal of Intellectual Disabilities. 2015;19(1):69-82. | Intervention |
| Crowley JJ. A clash of cultures: A&E and mental health. Accid Emerg Nurs. 2000;8(1):2-8. | Intervention |
| Kerker BD, Chor KHB, Hoagwood KE, Radigan M, Perkins MB, Setias J, et al. Detection and treatment of mental health issues by pediatric PCPs in New York state: An evaluation of project TEACH. Psychiatr Serv. 2015;66(4):430-3. | Intervention |
| Andrewes D, Say S, McLennan J. A self-administered computer-based educational program about eating disorder risk factors. Australian Psychologist. 1995;30(3):210-2. | Population |
| Beardslee WR, Avery MW, Ayoub C, Watts CL. Family Connections: Helping Early Head Start/Head Start Staff and Parents Address Mental Health Challenges. Zero to Three (J). 2009;29(6):34-43. | Population |
| Brown RT, Ahalt C, Steinman MA, Kruger K, Williams BA. Police on the front line of community geriatric health care: challenges and opportunities. Am Geriatr Soc. 2014;62(11):2191-8. | Population |
| Corrigan PW, Morris SB, Michaels PJ, Rafacz JD, Rusch N. Challenging the Public Stigma of Mental Illness: A Meta-Analysis of Outcome Studies. Psychiatr Serv. 2012;63(10):963-73. | Population |
| Graham H. Implementing integrated treatment for co-existing substance use and severe mental health problems in assertive outreach teams: training issues. Drug Alcohol Rev. 2004;23(4):463-70. | Population |
| Hassanein EE. Changing teachers' negative attitudes toward persons with intellectual disabilities. Behav Modif. 2015;39(3):367-89. | Population |
| Jorm AF, Kitchener BA, O'Kearney R, Dear KB. Mental health first aid training of the public in a rural area: a cluster randomized trial [ISRCTN53887541]. BMC psychiatry. 2004;4:33. | Population |
| McConkey R, McCormack B, Naughton M. Changing young people's perceptions of mentally handicapped adults. J Ment Defic Res. 1983;27:279-90. | Population |
| Petchers MK, Biegel DE, Drescher R. A video-based program to educate high school students about serious mental illness. Hosp Community Psychiatry. 1988;39(10):1102-3. | Population |
| Srikala B, Kishore Kumar KV. Empowering adolescents with life skills education in schools-School mental health program: Does it work. Indian Journal of Psychiatry. 2010;52(4):344-9. | Population |
| Van der Ploeg ES, Walker H, O'Connor DW. The feasibility of volunteers facilitating personalized activities for nursing home residents with dementia and agitation. Geriatr Nur (Lond). 2014;35(2):142-6. | Population |
| Wells K, Sherbourne C, Schoenbaum M, Ettner S, Duan N, Miranda J, et al. Five-year impact of quality improvement for depression: results of a group-level randomized controlled trial. Arch Gen Psychiatry. 2004;61(4):378-86. | Population |
| [No authors listed] Training police officers to help people with mental illnesses. J Psychosoc Nurs Ment Health Serv. 2001;39(6):10-1. | Study design |
| Adebowale V. Making progress. Mental Health Today. 2014:12-3. | Study design |
| Ahuja AS, Williams R. Involving patients and their carers in educating and training practitioners. Curr Opin Psychiatry. 2005;18(4):374-80. | Study design |
| Amorosi M. Depression: a preventive care between school and general population. Psychiatr Danub. 2010;22 Suppl 1:S126-31. | Study design |
| Andrick M. Offenders With Special Needs: Indiana's Approach to Preparing Staff to Meet the Challenge. Corrections Today. 2014;76(4):22-5. | Study design |
| Appelbaum KL. Police encounters with persons with mental illness: Introduction. J Am Acad Psychiatry Law. 2000;28(3):325. | Study design |
| Appleby L, Morriss R, Gask L, Roland M, Perry B, Lewis A, et al. An educational intervention for front-line health professionals in the assessment and management of suicidal patients (The STORM Project). Psychol Med. 2000;30(4):805-12. | Study design |
| Askell-Williams H, Lawson MJ, Murray-Harvey R. Teaching and learning about mental illnesses: An Australian perspective. International Journal of Mental Health Promotion. 2007;9(4):26-36. | Study design |
| Bahora M, Hanafi S, Chien VH, Compton MT. Preliminary evidence of effects of crisis intervention team training on self-efficacy and social distance. Administration & Policy in Mental Health. 2008;35(3):159-67. | Study design |
| Balogh R, Ouellette-Kuntz H, Bourne L, Lunsky Y, Colantonio A. Organising health care services for persons with an intellectual disability. Cochrane Database of Systematic Reviews 2008, Issue 4. Art.No.: CD007492. | Study design |
| Barcelos NM. The impact of crisis intervention team (cit) training on law enforcement officers in connecticut. Dissertation Abstracts International: Section B: The Sciences and Engineering. 2015;75(12-B(E)):No Pagination Specified. | Study design |
| Barker E, Kolves K, De Leo D. Management of suicidal and self-harming behaviors in prisons: systematic literature review of evidence-based activities. Archives of Suicide Research. 2014;18(3):227-40. | Study design |
| Beardslee WR, Ayoub C, Avery MW, Watts CL, O'Carroll KL. Family Connections: an approach for strengthening early care systems in facing depression and adversity. Am J Orthopsychiatry. 2010;80(4):482-95. | Study design |
| Beebee J. People with learning disabilities in the criminal justice system. Nurs Stand. 2010;24(38):35-8. | Study design |
| Berridge BJ, Hall K, Dillon P, Hides L, Lubman DI. MAKINGtheLINK: a school-based health promotion programme to increase help-seeking for cannabis and mental health issues among adolescents. Early intervention in psychiatry. 2011;5(1):81-8. | Study design |
| Bonfine N, Ritter C, Munetz MR. Police officer perceptions of the impact of Crisis Intervention Team (CIT) programs. International Journal of Law & Psychiatry. 2014;37(4):341-50. | Study design |
| Borum R, Deane MW, Steadman HJ, Morrissey J. Police perspectives on responding to mentally ill people in crisis: perceptions of program effectiveness. Behav Sci Law. 1998;16(4):393-405. | Study design |
| Borum R. Improving high risk encounters between people with mental illness and the police. J Am Acad Psychiatry Law. 2000;28(3):332-7. | Study design |
| Bostic JQ, Thurau L, Potter M, Drury SS. Policing the teen brain. Journal of the American Academy of Child & Adolescent Psychiatry. 2014;53(2):127-9. | Study design |
| Bowerman L, De Silva E, McMullen P, Clifford C. Suicide intervention training programmes improves the ability of first and second year paramedic students to identify and provide assistance to persons at risk. Australasian Journal of Paramedicine Conference. 2012;10(3). | Study design |
| Boyd E. Appropriate use of police officers? Psychiatr Serv. 2006;57(12):1811; author reply -2. | Study design |
| Broadbent M, Moxham L, Dwyer T. The development and use of mental health triage scales in Australia. International Journal of Mental Health Nursing. 2007;16(6):413-21. | Study design |
| Brown J, Wissow L, Cook B, Longway S, Caffery E, Pefaure C. Mental Health Communications Skills Training for Medical Assistants in Pediatric Primary Care. J Behav Health Serv Res. 2013;40(1):20-35. | Study design |
| Brownfield C, Eichinger L. Treating Offenders With Mental Illnesses in Iowa. Corrections Compendium. 2013;37(4):8-11. | Study design |
| Bruening MD. Effects of alternate format in-service delivery on teacher knowledge base and problem-solving related to autism & adaptations: What teachers need to know. Dissertation Abstracts International Section A: Humanities and Social Sciences. 2011;72(1-A):153. | Study design |
| Bynum R, Milan G, Phillips D, Weber B. Mental Health Education and Corrections. American Jails. 2008;22(4):23-9. | Study design |
| Callahan L. 'Correctional officer attitudes toward inmates with mental disorders.'. International Journal of Forensic Mental Health. 2004;3(1):37-54. | Study design |
| Camilleri P, McArthur M. Suicidal behaviour in prisons: learning from Australian and international experiences. International Journal of Law & Psychiatry. 2008;31(4):297-307. | Study design |
| Canada KE, Angell B, Watson AC. Intervening at the entry point: differences in how CIT trained and non-CIT trained officers describe responding to mental health-related calls. Community Ment Health J. 2012;48(6):746-55. | Study design |
| Cardinal JR. "Rethink autism": Effectiveness of web-based applied behavior video modeling program on the performance of paraeducators and students with autism spectrum disorder. Dissertation Abstracts International Section A: Humanities and Social Sciences. 2013;73(8-A(E)):No Pagination Specified. | Study design |
| Carrière GL. Linking women to health and wellness: Street Outreach takes a population health approach. Int J Drug Policy. 2008;19(3):205-10. | Study design |
| Carroll A, Forlin C, Jobling A. The Impact of Teacher Training in Special Education on the Attitudes of Australian Preservice General Educators towards People with Disabilities. Teacher Education Quarterly. 2003;30(3):65-79. | Study design |
| Chambers DW. Police work. J Am Coll Dent. 2009;76(4):2-3. | Study design |
| Chappell D, O'Brien A. Police responses to persons with a mental illness: international perspectives. International Journal of Law & Psychiatry. 2014;37(4):321-4. | Study design |
| Charette Y, Crocker AG, Billette I. Police encounters involving citizens with mental illness: use of resources and outcomes. Psychiatr Serv. 2014;65(4):511-6. | Study design |
| Charette Y, Crocker AG, Billette I. The judicious judicial dispositions juggle: characteristics of police interventions involving people with a mental illness. Can J Psychiatry. 2011;56(11):677-85. | Study design |
| Clark CA, Smith PR. Promoting Collaborative Practice for Children of Parents with Mental Illness and Their Families. Psychiatric Rehabilitation Journal. 2009;33(2):95-7. | Study design |
| Clarke A. Bridging the digital disconnect-using technology to support young people's mental health needs. Eur Child Adolesc Psychiatry. 2013;1):S195. | Study design |
| Clarke M, Jinks M, McMurran M. Readiness Enhancement Management Strategies (REMS): a proof of concept and evaluation feasibility study of staff training to improve service engagement by people with personality difficulties. J Psychiatr Ment Health Nurs. 2015;22(7):502-9. | Study design |
| Clayfield JC, Fletcher KE, Grudzinskas AJ, Jr. Development and validation of the Mental Health Attitude Survey for Police. Community Ment Health J. 2011;47(6):742-51. | Study design |
| Cochran S, Deane MW, Borum R. Improving police response to mentally ill people. Psychiatr Serv. 2000;51(10):1315-6. | Study design |
| Coggins MH, Pynchon MR. Mental health consultant to law enforcement: Secret Service development of a Mental Health Liaison Program. Behav Sci Law. 1998;16(4):407-22. | Study design |
| Compton MT, Bakeman R, Broussard B, Hankerson-Dyson D, Husbands L, Krishan S, et al. The police-based crisis intervention team (CIT) model: II. Effects on level of force and resolution, referral, and arrest. Psychiatr Serv. 2014;65(4):523-9. | Study design |
| Compton MT, Bakeman R, Broussard B, Hankerson-Dyson D, Husbands L, Krishan S, et al. The police-based crisis intervention team (CIT) model: I. Effects on officers' knowledge, attitudes, and skills. Psychiatr Serv. 2014;65(4):517-22. | Study design |
| Compton MT, Broussard B, Hankerson-Dyson D, Krishan S, Stewart-Hutto T. Do empathy and psychological mindedness affect police officers' decision to enter crisis intervention team training? Psychiatr Serv. 2011;62(6):632-8. | Study design |
| Compton MT, Chien VH. Factors related to knowledge retention after crisis intervention team training for police officers. Psychiatr Serv. 2008;59(9):1049-51. | Study design |
| Compton MT, Demir Neubert BN, Broussard B, McGriff JA, Morgan R, Oliva JR. Use of force preferences and perceived effectiveness of actions among Crisis Intervention Team (CIT) police officers and non-CIT officers in an escalating psychiatric crisis involving a subject with schizophrenia. Schizophr Bull. 2011;37(4):737-45. | Study design |
| Compton MT, Esterberg ML, McGee R, Kotwicki RJ, Oliva JR. Brief reports: crisis intervention team training: changes in knowledge, attitudes, and stigma related to schizophrenia. Psychiatr Serv. 2006;57(8):1199-202. | Study design |
| Compton MT, Quintero L, Esterberg ML. Assessing knowledge of schizophrenia: development and psychometric properties of a brief, multiple-choice knowledge test for use across various samples. Psychiatry Res. 2007;151(1-2):87-95. | Study design |
| Conner KR, Wood J, Pisani AR, Kemp J. Evaluation of a suicide prevention training curriculum for substance abuse treatment providers based on Treatment Improvement Protocol Number 50. J Subst Abuse Treat. 2013;44(1):13-6. | Study design |
| Cook BG, Rumrill PD, Beckett-Camarata J, Mitchell PR, Newman S, Sebaly KP, et al. The Impact of a Professional Development Institute on Faculty Members' Interactions with College Students with Learning Disabilities. Learning Disabilities: A Multidisciplinary Journal. 2006;14(1):67-76. | Study design |
| Coppens E, Van Audenhove C, Iddi S, Arensman E, Gottlebe K, Koburger N, et al. Effectiveness of community facilitator training in improving knowledge, attitudes, and confidence in relation to depression and suicidal behavior: results of the OSPI-Europe intervention in four European countries. J Affect Disord. 2014;165:142-50. | Study design |
| Cordner GW. A community policing approach to persons with mental illness. J Am Acad Psychiatry Law. 2000;28(3):326-31. | Study design |
| Crabtree J, Mack J. Developing champions to enhance the care of people with dementia in general hospitals. Nurs Times. 2010;106(48):13-4. | Study design |
| Cromby J, Harper D, Reavey P. Mental health teaching to UK psychology undergraduates: report of a survey. Journal of Community & Applied Social Psychology. 2008;18(1):83-90. | Study design |
| Cummings I, Jones S. Blue remembered skills: mental health awareness training for police officers. Journal of Adult Protection. 2010;12(3):14-9. | Study design |
| Davidson MW, Range LM. Are teachers of children and young adolescents responsive to suicide prevention training modules? Yes. Death Stud. 1999;23(1):61-71. | Study design |
| Davies J, Sampson M, Beesley F, Smith D, Baldwin V. An evaluation of Knowledge and Understanding Framework personality disorder awareness training: Can a co-production model be effective in a local NHS mental health Trust? Personality & Mental Health. 2014;8(2):161-8. | Study design |
| Davis JH. A Training Program Designed To Develop Knowledgeable Paraprofessionals with Improved Job Performance Skills To Meet the Needs of Teachers and Special Education Students 1995. | Study design |
| Dean K, Pinellas County School Board CFL, et al. We're Sold. Strategies for Overcoming Learning Differences. A Section 353 Training Project1995. | Study design |
| Dickstein LJ. Educational issues and innovative opportunities when integrating primary care and psychiatry: Training for 21st century competent, collaborative, medical professional development, part one: The need. Directions in Psychiatry. 2014;34(2):131-41. | Study design |
| Dixon VE. Development of Knowledge Domains and an Instrument to Assess Probation Officers' Knowledge of Offenders with Intellectual Disabilities: ProQuest LLC; 2011. | Study design |
| D'Souza CM, Forman SF, Austin SB. Follow-up evaluation of a high school eating disorders screening program: knowledge, awareness and self-referral. J Adolesc Health. 2005;36(3):208-13. | Study design |
| Eaton L. Charity calls for better training of police in handling mentally ill people. BMJ. 2008;337:a1531. | Study design |
| Ellis HA. Effects of a Crisis Intervention Team (CIT) training program upon police officers before and after Crisis Intervention Team training. Arch Psychiatr Nurs. 2014;28(1):10-6. | Study design |
| Ellis HA. The crisis intervention team: a revolutionary tool for law enforcement: the psychiatric-mental health nursing perspective. J Psychosoc Nurs Ment Health Serv. 2011;49(11):37-43; quiz 5. | Study design |
| El-Mallakh PL, Kiran K, El-Mallakh RS. Costs and savings associated with implementation of a police crisis intervention team. South Med J. 2014;107(6):391-5. | Study design |
| Evans CJ. Learning through Personal Interaction: Preparing Pre-Service Teachers for Students with Special Needs. Exceptionality Education Canada. 2004;14(2&3):189-208. | Study design |
| Fertman CI, Tarasevich SL. How Schools Address Students' Mental Health and Drug and Alcohol Concerns and Problems: Lessons from Student Assistance Programs. ERS Spectrum. 2004;22(2):35-46. | Study design |
| Fetherston AM, Sturmey P. The effects of behavioral skills training on instructor and learner behavior across responses and skill sets. Res Dev Disabil. 2014;35(2):541-62. | Study design |
| Franklin RE. Before the Bell Rings: The Importance of Preparing Pre-Service School Librarians to Serve Students with Special Needs. Knowledge Quest. 2011;39(3):58-63. | Study design |
| Freshwater D. Expanding roles in mental health care: the importance of training in prisons. Journal of Psychiatric & Mental Health Nursing. 2007;14(1):1-3. | Study design |
| Frierson RL. Commentary: Police officers and persons with mental illness. J Am Acad Psychiatry Law. 2013;41(3):356-8. | Study design |
| Fruhauf CA. Helping students understand aging and dementia: An innovative program. Dementia: The International Journal of Social Research and Practice. 2007;6(1):157-62. | Study design |
| Fyfe JJ. Policing the emotionally disturbed. J Am Acad Psychiatry Law. 2000;28(3):345-7. | Study design |
| Garcia CH, Oakes L, Alford C, Talamantes M. Evaluation of a culturally based training program for latino caregivers of dementia patients. Am Geriatr Soc. 2010;58:S222-S3. | Study design |
| Gask L, Lever-Green G, Hays R. Dissemination and implementation of suicide prevention training in one Scottish region. BMC Health Serv Res. 2008;8:246. | Study design |
| Geiman D. COTC/ACA Online Training. Corrections Today2014. p. 82-3. | Study design |
| Geiman D. Critical Training for Jail Personnel: Managing Inmates With Mental Illness. Corrections Today. 2010;72(2):22-. | Study design |
| Geiman D. Volunteers of America Indiana Implements Online Training. Corrections Today. 2012;74(2):16-7. | Study design |
| Gerdtz MF, Weiland TJ, Jelinek GA, Mackinlay C, Hill N. Perspectives of emergency department staff on the triage of mental health-related presentations: Implications for education, policy and practice. Emerg Med Australas. 2012;24(5):492-500. | Study design |
| Gibb BV. Mental Health First Aid for Public Safety - Three Case Studies. The Police Chief. 2014. p. 56-9. | Study design |
| Gittman E, Berger R. Impact of Teacher Education Courses on Paraprofessionals' Job Performance, Knowledge, and Goals. 1997. | Study design |
| Gleason MM, Heller SS, Nagle GA, Boothe A, Keyes A, Rice J. Mental Health Screening in Child Care: Impact of a Statewide Training Session. Early Childhood Research & Practice. 2012;14(2). | Study design |
| Glickman S, Bachman M, Williams J, Sheitman B, Steiner B, Brice J, et al. An advanced practice paramedic program can safely and effectively divert acute mental health patients from the ED to a community mental health center. Acad Emerg Med. 2014;1):S25-S6. | Study design |
| Glynn SM, Randolph ET, Garrick T, Lui A. A Proof of Concept Trial of an Online Psychoeducational Program for Relatives of Both Veterans and Civilians Living with Schizophrenia. Psychiatric Rehabilitation Journal. 2010;33(4):278-87. | Study design |
| Gotto J, Mayorga L. Enhancing psychiatric triage by developing and implementing interactive innovative education modules for social workers via the web. Psychooncology. 2011;20:204-5. | Study design |
| Hanover NH. PoliceCommunity.net online training program. Law & Order. 2011;59(2):8. | Study design |
| Hatfield RE. Training law enforcement in mental health: A broad-based model. Dissertation Abstracts International: Section B: The Sciences and Engineering. 2014;74(9-B(E)):No Pagination Specified. | Study design |
| Hawkins JD, Kosterman R, Catalano RF, Hill KG, Abbott RD. Promoting positive adult functioning through social development intervention in childhood: long-term effects from the Seattle Social Development Project.[Erratum appears in Arch Pediatr Adolesc Med. 2005 May;159(5):469]. Arch Pediatr Adolesc Med. 2005;159(1):25-31. | Study design |
| Hayes AJ, Shaw JJ, Lever-Green G, Parker D, Gask L. Improvements to suicide prevention training for prison staff in England and Wales. Suicide Life Threat Behav. 2008;38(6):708-13. | Study design |
| Health Education England. E-learning course on dementia for care workers and assistants. Trimbos Quarterly. 2013:123:5. | Study design |
| Hemmings A. Mental health. Calls for help. The Health service journal. 1997;107(5557):34-5. | Study design |
| Henderson CA. Preparing college faculty for working with students with asperger's syndrome: A web-based training module. Dissertation Abstracts International Section A: Humanities and Social Sciences. 2013;73(12-A(E)):No Pagination Specified. | Study design |
| Hides L, Lubman DI, Elkins K, Catania LS, Rogers N. Feasibility and acceptability of a mental health screening tool and training programme in the youth alcohol and other drug (AOD) sector. Drug Alcohol Rev. 2007;26(5):509-15. | Study design |
| Hills DJ, Robinson T, Kelly B, Heathcote S. Outcomes from the trial implementation of a multidisciplinary online learning program in rural mental health emergency care. Education for Health. 2010;23(1):351. | Study design |
| Hollander Y, Lee SJ, Tahtalian S, Young D, Kulkarni J. Challenges Relating to the Interface Between Crisis Mental Health Clinicians and Police When Engaging with People with a Mental Illness. Psychiatry, Psychology and Law. 2012;19(3):402-11. | Study design |
| Hughes M, Miami Univ CGFLSoE. Project Bridge: Preparing African-American Teachers To Work with Young Children with Disabilities and Their Families. Final Report. 2001. | Study design |
| Husted JR, Charter RA, Perrou B. California law enforcement agencies and the mentally ill offender. Bulletin of the American Academy of Psychiatry & the Law. 1995;23(3):315-29. | Study design |
| Igric L. Improvement of teacher attitudes toward pupils with special needs through a teacher-training programme. J Intellect Disabil Res. 2000;44:330-. | Study design |
| James RK, Crews W, Gilliland BE. Systems consultation: Working with a metropolitan police department. Dougherty, A Michael [Ed] (1995) Case studies in human services consultation (pp 133-154) 193 pp Belmont, CA, US: Thomson Brooks/Cole Publishing Co; US. 1995:133-54. | Study design |
| Johnson NL, Lashley J, Stonek AV, Bonjour A. Children with developmental disabilities at a pediatric hospital: staff education to prevent and manage challenging behaviors. J Pediatr Nurs. 2012;27(6):742-9. | Study design |
| Junco R, Salter DW. Improving the Campus Climate for Students with Disabilities through the Use of Online Training. NASPA Journal. 2004;41(2):263-76. | Study design |
| Kent J, Gunasekaran S. Mentally disordered detainees in the police station: The role of the psychiatrist. Advances in Psychiatric Treatment. 2010;16(2):115-23. | Study design |
| King DL, Kalucy RS, De Crespigny CF, Stuhlmiller CM, Thomas LJ. Mental health and alcohol and other drug training for emergency department workers: one solution to help manage increasing demand. Emerg Med Australas. 2004;16(2):155-60. | Study design |
| Klassen L, Fallu A. LINK: The adult Attention-Deficit/Hyperactivity Disorder programme (ADHD): Connecting - Educating - Advancing (Part II). ADHD Attention Deficit and Hyperactivity Disorders. 2015;7:S58. | Study design |
| Krameddine YI, Demarco D, Hassel R, Silverstone PH. A Novel Training Program for Police Officers that Improves Interactions with Mentally Ill Individuals and is Cost-Effective. Frontiers in psychiatry Frontiers Research Foundation. 2013;4:9. | Study design |
| Krameddine YI, Silverstone PH. How to improve interactions between police and the mentally ill. Frontiers in Psychiatry. 2015;6(JAN). | Study design |
| Kroening K. Measuring outcomes: The impact of three day mental health training on juvenile corrections officers day to day decision making regarding the mentally ill youth in their care. Adm Policy Ment Health. 2004;31(5):431. | Study design |
| Kutcher S, Wei Y, McLuckie A, Bullock L. Educator mental health literacy: A programme evaluation of the teacher training education on the mental health & high school curriculum guide. Advances in School Mental Health Promotion. 2013;6(2):83-93. | Study design |
| Kuwent SM. Education and management of borderline adolescents in special education settings: A special education teacher training program. Dissertation Abstracts International: Section B: The Sciences and Engineering. 1995;56(5-B):2871. | Study design |
| Lacey P. Interdisciplinary training for staff working with people with profound and multiple learning disabilities. J Interprof Care. 1998;12(1):43-52. | Study design |
| Langeveld J, Joa I, Larsen TK, Rennan JA, Cosmovici E, Johannessen JO. Teachers' awareness for psychotic symptoms in secondary school: the effects of an early detection programme and information campaign. Early intervention in psychiatry. 2011;5(2):115-21. | Study design |
| Lary DB. The effect of an educational program for direct care workers on attitude of stigma toward mentally ill youth in a juvenile justice system. Dissertation Abstracts International: Section B: The Sciences and Engineering. 2007;68(6-B):3693. | Study design |
| Lavigne JE, King DA, Lu N, Knox KL, Kemp JE. Pharmacist and pharmacy staff knowledge and attitudes towards suicide and suicide prevention after a national va training program. Value Health. 2011;14 (3):A199-A200. | Study design |
| Lee N, Jenner L, Baker A, Ritter A, Hides L, Norman J, et al. Screening and intervention for mental health problems in alcohol and other drug settings: Can training change practitioner behaviour? Drugs (Abingdon Engl). 2011;18(2):157-60. | Study design |
| Leppert T, Probst P. Development and evaluation of a psychoeducative group training program for teachers of autistic students with mental retardation. Z Kinder Jugendpsychiatr Psychother. 2005;33(1):49-58. | Study design |
| Lerman DC, Tetreault A, Hovanetz A, Strobel M, Garro J. Further evaluation of a brief, intensive teacher-training model. J Appl Behav Anal. 2008;41(2):243-8. | Study design |
| Ljiljana I. Improvement of the attitudes of teachers toward pupils with special needs through a teacher training programme. J Intellect Disabil Res. 2000;44:369-70. | Study design |
| Loue S, Lloyd LS. Training program in mental illness for immigration officers. Psychiatr Serv. 2005;56(11):1458. | Study design |
| Lurigio AJ, Smith A, Harris A. The Challenge of Responding to People with Mental Illness: Police Officer Training and Special Programmes. Police Journal. 2008;81(4):295-322. | Study design |
| Lynch TF, Horton R, Wynne MM. Taking the Trauma Out of Security. Security Management. 2009;53(7):48-54. | Study design |
| Maggio E, Health R. Making It Easier for School Staff to Help Traumatized Students. Research Highlights. RAND Corporation, 2009. | Study design |
| Maltman L, Hamilton L. Preliminary evaluation of personality disorder awareness workshops for prison staff. The British Journal of Forensic Practice. 2011;13(4):244-56. | Study design |
| Marotta P, Barnum J, Watson A, Caplan J. Crisis Intervention Team Training Programs for Law Enforcement Officers: A Systematic Review. Campbell Systematic Reviews2014. | Study design |
| Matthieu MM, Ross A, Knox KL. Program Evaluation of the Samaritans of New York's Public Education Suicide Awareness and Prevention Training Program. Brief Treatment & Crisis Intervention. 2006;6(4):295-307. | Study design |
| Mayall E, Oathamshaw S, Lovell K, Pusey H. Development and piloting of a multidisciplinary training course for detecting and managing depression in the older person. Journal of Psychiatric & Mental Health Nursing. 2004;11(2):165-71. | Study design |
| McGonigle JJ, Migyanka JM, Glor-Scheib SJ, Cramer R, Fratangeli JJ, Hegde GG, et al. Development and evaluation of educational materials for pre-hospital and emergency department personnel on the care of patients with autism spectrum disorder. Journal of Autism & Developmental Disorders. 2014;44(5):1252-9. | Study design |
| McPherson-Sexton S, Hostetler B. How to Respond to the Crisis Victim with PTSD Symptoms: An Intervener's Guide. Journal of Police Crisis Negotiations. 2009;9(1):61-6. | Study design |
| Meoli M, Rathbun D. Being ready to deploy. Interoperable core skills for training to respond to violent incidents. Journal of Emergency Medical Services. 2014;Suppl:34-6, 8-40. | Study design |
| Minoudis P, Craissati J, Shaw J, McMurran M, Freestone M, Chuan SJ, et al. An evaluation of case formulation training and consultation with probation officers. Criminal Behaviour & Mental Health. 2013;23(4):252-62. | Study design |
| Mishara BL, Martin N. Effects of a comprehensive police suicide prevention program. Crisis: Journal of Crisis Intervention & Suicide. 2012;33(3):162-8. | Study design |
| Mitchell AJ, Dennis M. Self harm and attempted suicide in adults: 10 practical questions and answers for emergency department staff. Emerg Med J. 2006;23(4):251-5. | Study design |
| Moor S, Sharrock G, Scott J, McQueen H, Wrate R, Cowan J, et al. Evaluation of a teaching package designed to improve teachers' recognition of depressed pupils-a pilot study. J Adolesc. 2000;23(3):331-42. | Study design |
| Morriss R, Gask L, Battersby L, Francheschini A, Robson M. Teaching front-line health and voluntary workers to assess and manage suicidal patients. J Affect Disord. 1999;52(1-3):77-83. | Study design |
| Morrissey JP, Fagan JA, Cocozza JJ. New models of collaboration between criminal justice and mental health systems. Am J Psychiatry. 2009;166(11):1211-4. | Study design |
| Murray C, Lombardi A, Wren CT. The Effects of Disability-Focused Training on the Attitudes and Perceptions of University Staff. Remedial and Special Education. 2011;32(4):290-300. | Study design |
| Naismith SL, Hickie IB, Scott EM, Davenport TA. Effects of mental health training and clinical audit on general practitioners' management of common mental disorders. Med J Aust. 2001;175 Suppl:S42-7. | Study design |
| Nash T, Norwich B. The initial training of teachers to teach children with special educational needs: A national survey of English Post Graduate Certificate of Education programmes. Teaching and Teacher Education. 2010;26(7):1471-80. | Study design |
| National Commission on Correctional Health C. Position statement: prevention of juvenile suicide in correctional settings. Journal of Correctional Health Care. 2009;15(3):227-31. | Study design |
| Nigro-Bruzzi D. The effects of pyramidal training on staff behavior and manding in children with autism. Dissertation Abstracts International: Section B: The Sciences and Engineering. 2011;71(8-B):5107. | Study design |
| Noga HL, Walsh EC, Shaw JJ, Senior J. The development of a mental health screening tool and referral pathway for police custody. Eur J Public Health. 2015;25(2):237-42. | Study design |
| Norwich B, Nash T. Preparing Teachers to Teach Children with Special Educational Needs and Disabilities: The Significance of a National PGCE Development and Evaluation Project for Inclusive Teacher Education. Journal of Research in Special Educational Needs. 2011;11(1):2-11. | Study design |
| Oliva JR, Compton MT. A statewide Crisis Intervention Team (CIT) initiative: evolution of the Georgia CIT program. J Am Acad Psychiatry Law. 2008;36(1):38-46. | Study design |
| Oordt MS, Jobes DA, Fonseca VP, Schmidt SM. Training Mental Health Professionals to Assess and Manage Suicidal Behavior: Can Provider Confidence and Practice Behaviors be Altered? Suicide Life Threat Behav. 2009;39(1):21-32. | Study design |
| Parker GF. Impact of a mental health training course for correctional officers on a special housing unit. Psychiatr Serv. 2009;60(5):640-5. | Study design |
| Pearce J, Pearce J, Mann MK, Jones C, Holmsten S, Olff M, et al. Train-The-Trainers Programmes for Improving Clinical Behaviour in Health and Social Care Professionals. Campbell Systematic Reviews2011. | Study design |
| Perske R. Understanding persons with intellectual disabilities in the criminal justice system: indicators of progress? Ment Retard. 2004;42(6):484-7. | Study design |
| Peterson P, Showalter S. Meeting the Need for Special Education Teachers for Culturally Linguistically Diverse Students with Disabilities. Journal of College Teaching & Learning. 2010;7(10):7-10. | Study design |
| Pickett SA, Diehl S, Steigman PJ, Prater JD, Fox A, Cook JA. Early outcomes and lessons learned from a study of the Building Recovery of Individual Dreams and Goals through Education and Support (BRIDGES) program in Tennessee. Psychiatric Rehabilitation Journal. 2010;34(2):96-103. | Study design |
| Pinfold V, Huxley P, Thornicroft G, Farmer P, Toulmin H, Graham T. Reducing psychiatric stigma and discrimination--evaluating an educational intervention with the police force in England. Soc Psychiatry Psychiatr Epidemiol. 2003;38(6):337-44. | Study design |
| Pinfold V, Thornicroft G, Huxley P, Farmer P. Active ingredients in anti-stigma programmes in mental health. Int Rev Psychiatry. 2005;17(2):123-31. | Study design |
| Podhajski B, Stern Center for L, Learning WVT. Teaching Adults with Learning Disabilities. A Model Training Program for ABE Tutors. 1995. | Study design |
| Poland AL. Got training? The effect of mental health training on the attitudes and behaviors of direct care workers in a residential facility for juvenile offenders. Dissertation Abstracts International Section A: Humanities and Social Sciences. 2010;70(9-A):3647. | Study design |
| Pollard JS, Higbee TS, Akers JS, Brodhead MT. An evaluation of interactive computer training to teach instructors to implement discrete trials with children with autism. J Appl Behav Anal. 2014;47(4):765-76. | Study design |
| Pollock WM. The Impact of On-Line Training on College Faculty Attitudes and Knowledge of Students with Disabilities: ProQuest LLC; 2009. | Study design |
| Pomeroy EC, Parrish DE. Online training on fetal alcohol spectrum disorders for court-appointed special advocates volunteers. Health Soc Work. 2013;38(3):159-66. | Study design |
| Pompili M, Lester D, Innamorati M, Del Casale A, Girardi P, Ferracuti S, et al. Preventing suicide in jails and prisons: suggestions from experience with psychiatric inpatients. J Forensic Sci. 2009;54(5):1155-62. | Study design |
| Porter J. A training program to address the management of students with PTSD symptoms in a residential vocational and academic setting. Dissertation Abstracts International: Section B: The Sciences and Engineering. 2010;70(8-B):5180. | Study design |
| Portland State Univ ORR, Training Center on Family S, Children's Mental H, University of South Florida TR, Training Center for Children's Mental H. Staff Perspectives on Consultation and Integrated Mental Health Services in Early Childhood Settings. Data Trends #110. Research and Training Center on Family Support and Children's Mental Health, 2005. | Study design |
| Powers JD. Scientifically Supported Mental Health Intervention in Schools: Meeting Accountability Demands with an Online Resource. Journal of Evidence-Based Social Work. 2012;9(3):231-40. | Study design |
| Premalatha Chinnayya H, Chandrashekar CR, Moily S, Raghuram A, Subramanya KR, Shanmugham V, et al. Training Primary Care Health Workers in Mental Health Evaluation of Attitudes towards Mental Illness before and after Training. Int J Soc Psychiatry. 1990;36(4):300-7. | Study design |
| Probst P, Leppert T. Brief report: outcomes of a teacher training program for autism spectrum disorders. Journal of Autism & Developmental Disorders. 2008;38(9):1791-6. | Study design |
| Pyke J, Butterill D. A WORKSHOP FOR RECEPTIONISTS IN MENTAL HEALTH SETTINGS. Psychiatric Rehabilitation Journal. 2001;24(4):401-4. | Study design |
| Qureshi NA, Al-Ghamdy YS, Al-Haddad NS, Abdelgadir MH, Tawfik MH. Integration of mental health care into primary care. Preliminary observations of continuing implementation phase. Saudi Med J. 2001;22(10):899-906. | Study design |
| Rae H, McKenzie K, Murray G. The impact of training on teacher knowledge about children with an intellectual disability. Journal of Intellectual Disabilities. 2011;15(1):21-30. | Study design |
| Reeves S PL, Goldman J, Freeth D, ZwarensteinM. Interprofessional education: effects on professional practice and healthcare outcomes (update) (Review). Cochrane Database of Systematic Reviews 2013, Issue 3 Art No: CD002213 DOI: 101002/14651858CD002213pub3. | Study design |
| Reis C, Cornell D. An Evaluation of Suicide Gatekeeper Training for School Counselors and Teachers. Professional School Counseling. 2008;11(6):386-94. | Study design |
| Reutzel TJ, Desai A, Workman G, Atkin JA, Grady S, Todd T, et al. Medication management in primary and secondary schools: evaluation of mental health related in-service education in local schools. J Sch Nurs. 2008;24(4):239-48. | Study design |
| Robbins LA. Which is more effective in educating teachers to work with children with autism: An online or face-to-face format of instruction? Dissertation Abstracts International Section A: Humanities and Social Sciences. 2011;71(10-A):3620. | Study design |
| Robertson K, Elcock S, Milburn C, Annesley P, Jones J, Völlm BA. An evaluation of the staff training within the trauma and self injury (TASI) programme in the National High Secure Healthcare Service for Women (NHSHSW). Journal of Forensic Practice. 2013;15(2):141-50. | Study design |
| Robinson T, Hills D, Rossiter R. Mental health emergency care in Australia: an educational program for clinicians. Education for Health. 2013;26(3):172-7. | Study design |
| Sage R. Communicating with students who have learning and behaviour difficulties: A continuing professional development programme. Emotional and Behavioural Difficulties. 2005;10(4):281-97. | Study design |
| Schein RB. Mental health and mental illness in adolescence: A training program for youth workers. Dissertation Abstracts International: Section B: The Sciences and Engineering. 2013;74(5-B(E)):No Pagination Specified. | Study design |
| Scoville D. You Are Not a Psychologist. Police. 2012;36(3):45-9. | Study design |
| Sexton E, Ryst E, Gardner J, Bennett K. Effective practice in an ever-shifting landscape: A multidisciplinary approach to behavioral and mental health support in schools. Advances in School Mental Health Promotion. 2011;4(4):22-34. | Study design |
| Shanley C, Quirke S, Shaw L, Sammut A. Working with organizations to implement dementia awareness training for public contact staff. American Journal of Alzheimer's Disease & Other Dementias. 2004;19(3):166-71. | Study design |
| Sharp K. Patrol paradigm shift: cit and the mentally ill. Law & Order. 2012;60(1):48-51. | Study design |
| Shaw K. NC Prison Focuses on Treatment for Mentally Ill Inmates. Corrections Today. 2013;75(3):12-. | Study design |
| Shim RS, Compton MT. Pilot testing and preliminary evaluation of a suicide prevention education program for emergency department personnel. Community Ment Health J. 2010;46(6):585-90. | Study design |
| Shrubb R. Safety first. Mental Health Today. 2008:15-7. | Study design |
| Silver T. In-service training of consumer staff member in mental health and vocational services. Psychiatric Rehabilitation Journal. 1998;21(3):284. | Study design |
| Silverstone PH, Krameddine YI, DeMarco D, Hassel R. A novel approach to training police officers to interact with individuals who may have a psychiatric disorder. J Am Acad Psychiatry Law. 2013;41(3):344-55. | Study design |
| Smart D, Pollard C, Walpole B. Mental health triage in emergency medicine. Aust N Z J Psychiatry. 1999;33(1):57-69. | Study design |
| Smidt A, Balandin S, Reed V, Sigafoos J. A Communication Training Programme for Residential Staff Working with Adults with Challenging Behaviour: Pilot Data on Intervention Effects. Journal of Applied Research in Intellectual Disabilities. 2007;20(1):16-29. | Study design |
| Smoyak SA. Pennsylvania police officer trained for mental illness crises. J Psychosoc Nurs Ment Health Serv. 2013;51(9):9-10. | Study design |
| Sorkin CB, Roane DM, Pollack MH, Blank L, Serby MJ. The manhattan coalition of mental health issues of the elderly: Evaluation of "it's not just the blues" training program for culturally diverse home attendants caring for older adults. Am J Geriatr Psychiatry. 2012;1):S101-S2. | Study design |
| Staley GM. Development of a web-based officer's field guide to mental illness. Dissertation Abstracts International Section A: Humanities and Social Sciences. 2012;73(5-A):1640. | Study design |
| Stewart C. Police intervention in mental health crisis: A case study of the Bloomington Crisis Intervention Team (CIT) program. Dissertation Abstracts International Section A: Humanities and Social Sciences. 2010;71(2-A):723. | Study design |
| Suhrheinrich J, Stahmer AC, Reed S, Schreibman L, Reisinger E, Mandell D. Implementation challenges in translating pivotal response training into community settings. Journal of Autism & Developmental Disorders. 2013;43(12):2970-6. | Study design |
| Taliaferro AR, Hammond L, Wyant K. Preservice physical educators' self-efficacy beliefs toward inclusion: the impact of coursework and practicum. Adapted Physical Activity Quarterly. 2015;32(1):49-67. | Study design |
| Tarren-Sweeney M, Carr V. Principles for development of multi-disciplinary, mental health learning modules for undergraduate, postgraduate and continuing education. Education for Health. 2004;17(2):204-12. | Study design |
| Taylor J. The great training robbery. Nurs Times. 2008;104(14):20-1. | Study design |
| Teller JL, Munetz MR, Gil KM, Ritter C. Crisis intervention team training for police officers responding to mental disturbance calls. Psychiatr Serv. 2006;57(2):232-7. | Study design |
| Trent SC, Driver BL, Wood MH, Parrott PS, Martin TF, Smith WG. Creating and sustaining a special education/general education partnership: a story of change and uncertainty. Teaching and Teacher Education. 2003;19(2):203-19. | Study design |
| Turner A, Glantz K, Gall J. A Practitioner-Researcher Partnership to Develop and Deliver Operational Value of Threat, Risk and Vulnerability Assessment Training to meet the Requirements of Emergency Responders. Journal of Homeland Security & Emergency Management. 2012;9(2):-1. | Study design |
| Turner A, Glantz K, Gall J. A Practitioner-Researcher Partnership to Develop and Deliver Operational Value of Threat, Risk and Vulnerability Assessment Training to meet the Requirements of Emergency Responders. Journal of Homeland Security & Emergency Management. 2013;10(1):1-14. | Study design |
| Unger KV, Pfaltzgraf B, Nikkel RE. A supported education program in a state psychiatric hospital. Psychiatr Serv. 2010;61(6):632. | Study design |
| Vamvakas A, Rowe M. Mental health training in emergency homeless shelters. Community Ment Health J. 2001;37(3):287-95. | Study design |
| van den Brink RH, Broer J, Tholen AJ, Winthorst WH, Visser E, Wiersma D. Role of the police in linking individuals experiencing mental health crises with mental health services. BMC Psychiatry. 2012;12(1):1-7. | Study design |
| Vermette HS, Pinals DA, Appelbaum PS. Mental health training for law enforcement professionals. J Am Acad Psychiatry Law. 2005;33(1):42-6. | Study design |
| Voss S, Benger J, Black S, Cheston R, Cullum S, Purdy S, et al. Training on dementia for emergency ambulance staff: Research agenda and opportunities. Int J Geriatr Psychiatry. 2015;30(5):549-50. | Study design |
| Wahowiak L. Law, order, and lows. Diabetes Forecast. 2014;67(1):22, 4-5. | Study design |
| Walker H, Young J, Langton D, Thomson L. Organisational impact of a forensic education programme. Journal of Forensic Practice. 2013;15(3):218-30. | Study design |
| Watson AC, Corrigan PW, Ottati V. Police officers' attitudes toward and decisions about persons with mental illness. Psychiatr Serv. 2004;55(1):49-53. | Study design |
| Watson AC, Morabito MS, Draine J, Ottati V. Improving police response to persons with mental illness: a multi-level conceptualization of CIT. International Journal of Law & Psychiatry. 2008;31(4):359-68. | Study design |
| Watson AC, Swartz J, Bohrman C, Kriegel LS, Draine J. Understanding how police officers think about mental/emotional disturbance calls. International Journal of Law & Psychiatry. 2014;37(4):351-8. | Study design |
| Weaver CM, Joseph D, Dongon SN, Fairweather A, Ruzek JI. Enhancing services response to crisis incidents involving veterans: a role for law enforcement and mental health collaboration. Psychological Services. 2013;10(1):66-72. | Study design |
| Wells W, Schafer JA. Officer perceptions of police responses to persons with a mental illness. Policing. 2006;29(4):578-601. | Study design |
| White SW, Sukhodolsky DG, Rains AL, Foster D, McGuire JF, Scahill L. Elementary school teachers' knowledge of Tourette syndrome, Obsessive-Compulsive Disorder, & Attention Deficit/Hyperactivity Disorder: Effects of teacher training. Journal of Developmental and Physical Disabilities. 2011;23(1):5-14. | Study design |
| Wilcox N. FBI - The Importance of Mental Health Training in Law Enforcement. FBI Law Enforcement Bulletin. 2015:18-22. | Study design |
| Wilkniss S, Murphy J, Fitzgerald K, Szalai-Raymond M. Learning from the 'drama' of police encounters. Role-playing activities help police prepare for helping mental health consumers in crisis. Behavioral Healthcare. 2007;27(10):27-30. | Study design |
| Williams R. Addressing Mental Health in the Justice System. Ncsl Legisbrief. 2015;23(31):1-2. | Study design |
| Willner P, Bridle J, Price V, Dymond S, Lewis G. What do NHS staff learn from training on the Mental Capacity Act (2005)? Legal & Criminological Psychology. 2013;18(1):83-101. | Study design |
| Wise J. People in mental health crises are treated like criminals, says report. BMJ. 2013;346:f4036. | Study design |
| Wyn J, Cahill H, Holdsworth R, Rowling L, Carson S. MindMatters, a whole-school approach promoting mental health and wellbeing. Aust N Z J Psychiatry. 2000;34(4):594-601. | Study design |
| Yates P, Kramer T, Garralda ME. Use of a Routine Mental Health Measure in an Adolescent Secure Unit. Br J Psychiatry. 2006;188:583-4. | Study design |
| Zolezzi M, Blake A. Principles-based learning design for an online postgraduate psychiatric pharmacy course. Am J Pharm Educ. 2008;72(5):107 | Study design |
