# Supplementary material for: Mental health training programmes for non-mental health trained professionals coming into contact with people with mental ill health: a systematic review of effectiveness
Source: BMC Psychiatry. 2017 May 25;17:196. doi: 10.1186/s12888-017-1356-5 (PMC5445268; doi:10.1186/s12888-017-1356-5)
Supplement: Supplementary file 3 — Quality assessments. (DOCX 19 kb) [file 12888_2017_1356_MOESM3_ESM.docx]

**Additional file 3: Quality assessments**

**A: Risk of bias assessment of systematic review using ROBIS tool [9]**

| **Study** | **Risk of bias** | **Observations** |
| --- | --- | --- |
| Compton (2008) [13] | High | There were concerns in all areas of the assessment. Review methods were not addressed. While the discussion included recognition of the limitations of the identified studies in supporting roll out of CIT, the results of all the papers were presented in a positive way. |

**B: Risk of bias assessment of RCTs using the Cochrane Risk of Bias tool for RCTs [10]**

| **Study** | **Random sequence generation** | **Allocation concealment** | **Blinding of participants and personnel** | **Blinding of outcome assessment** | **Incomplete outcome data** | **Selective outcome reporting** | **Summary of assessment** |
| --- | --- | --- | --- | --- | --- | --- | --- |
| Dorsey et al (2012) [25] | Unclear | Low | Unclear | Low | High | Low | High risk of bias |
| Hart & More (2013) [24] | Unclear | Low | Unclear | Unclear | Unclear | Unclear | Unclear risk of bias |
| Jorm et al (2010) [23] | High | Low | Low | Unclear | High | Low | High risk of bias |
| Kolko et al (2012) [22] | Unclear | Unclear | Low | Low | Low | Unclear | Unclear risk of bias |
| Lipson et al (2014) [21] | Low | High | High | High | High | Low | High risk of bias |
| McVey et al (2008) [20] | Unclear | Unclear | Unclear | Unclear | Unclear | Low | Unclear risk of bias |
| Moor et al (2007 [19] | Unclear | Unclear | Low | Unclear | Low | Low | Unclear risk of bias |
| Ostberg & Rydell (2012) [18] | Unclear | Unclear | High | Unclear | High | Low | High risk of bias |
| Rafacz (2012) [17] | Low | Unclear | Unclear | Unclear | Low | Low | Unclear risk of bias |
| Svensson & Hansson (2014) [16] | Low | Unclear | Low | Unclear | High | Low | High risk of bias |
| Thombs et al (2015) [14] | Unclear | High | Unclear | Unclear | Unclear | High | High risk of bias |
| Teagardin et al (2012) [15] | Unclear | High | Unclear | Unclear | Unclear | Unclear | High risk of bias |

**C: Risk of bias assessment of non-randomised controlled trials using the Newcastle-Ottawa Quality Assessment Scale for Cohort Studies [11]**

| **Study ID** | **Selection** | | | | **Comparability** | | **Outcome** | | | |
| --- | --- | --- | --- | --- | --- | --- | --- | --- | --- | --- |
|  | **Representative-ness of the exposed cohort** | **Selection of the non-exposed cohort** | **Ascertainment of exposure** | **Demonstration that outcome of interest was not present at start of study** | **Comparability of cohorts on the basis of the design or analysis** | | **Assessment of outcome** | **Was follow-up long enough for outcomes to occur** | **Adequacy of follow up of cohorts** | |
| Bailey et al (2001) [26] | ++  Somewhat representative of the average trainee police officer in the community | +  Drawn from the same community as the exposed cohort | Not clear when or how surveys were administered | Unclear | +  Study controls for pre-existing knowledge and skills | No other factors controlled for | Survey – self allocated ‘code’ names | No: follow up was immediately post training only | | ++  Subjects lost to follow up unlikely to introduce bias - small number lost - > 80 % |
| Hansson & Markstrom (2014) [27] | +  Somewhat representative of the average student police officer in the community | +  Drawn from the same community as the exposed cohort | +  Secure record: completion of survey in classroom pre and post | +  Yes - participants familiarity with mental illness assessed at base line | No control for pre-existing knowledge and skills | No other factors controlled for | Self report | No: only intervention group followed up at 6 months | | +  Subjects lost to follow up unlikely to introduce bias - small number lost - > 80 % |
| Herrington & Pope (2013) [28] | +  Somewhat representative of the average police officer in the community | +  Drawn from the same community as the exposed cohort | No description | Not reported | No control for pre-existing knowledge and skills | No other factors controlled for | Not reported | +  Yes: 18 months | | Follow up rate varies and overall insufficient data reported |

**D: Risk of bias assessment of non-comparative studies using the National Institutes for Health tool for studies without a control group [12]**

| **Study** | **Quality Rating (Good, Fair, or Poor)** | **Observations** |
| --- | --- | --- |
| Forni et al (2009) [29] | Fair | Reasonably well reported evaluation consisted of immediate post training questionnaire on presentation and content only 280/364 completed forms. However, unclear if pre-specified and validity/reliability of the questionnaire uncertain. Little detail on the evaluation form or how it was created. The findings of the evaluation are specific to the setting and participants and are not generalizable, however interesting and fair report on local initiative. |
| Norris & Cooke (2000) [30] | Fair | Fair level of detail about training given but evaluation was immediate feedback and then retrospective survey so no baseline data collected. 43% response rate (55/132). The findings of the evaluation are specific to the setting and participants and are not generalizable. Interesting and fair report on local initiative. Little detail of survey content. |
| Pinfold et al (2003) [31] | Good | Well reported pre and post evaluation |
